# A Multivariate Granger Causality Concept towards Full Brain Functional Connectivity

**DOI:** 10.1371/journal.pone.0153105

**Published:** 2016-04-11

**Authors:** Christoph Schmidt, Britta Pester, Nicole Schmid-Hertel, Herbert Witte, Axel Wismüller, Lutz Leistritz

**Affiliations:** 1 Institute of Medical Statistics, Computer Sciences and Documentation, Jena University Hospital, Friedrich Schiller University, Jena, Germany; 2 Institute of Anatomy I, Jena University Hospital, Friedrich Schiller University, Jena, Germany; 3 Department of Imaging Sciences, Department of Biomedical Engineering, University of Rochester Medical Center, Rochester, New York, United States of America; Indiana University, UNITED STATES

## Abstract

Detecting changes of spatially high-resolution functional connectivity patterns in the brain is crucial for improving the fundamental understanding of brain function in both health and disease, yet still poses one of the biggest challenges in computational neuroscience. Currently, classical multivariate Granger Causality analyses of directed interactions between single process components in coupled systems are commonly restricted to spatially low- dimensional data, which requires a pre-selection or aggregation of time series as a preprocessing step. In this paper we propose a new fully multivariate Granger Causality approach with embedded dimension reduction that makes it possible to obtain a representation of functional connectivity for spatially high-dimensional data. The resulting functional connectivity networks may consist of several thousand vertices and thus contain more detailed information compared to connectivity networks obtained from approaches based on particular regions of interest. Our large scale Granger Causality approach is applied to synthetic and resting state fMRI data with a focus on how well network community structure, which represents a functional segmentation of the network, is preserved. It is demonstrated that a number of different community detection algorithms, which utilize a variety of algorithmic strategies and exploit topological features differently, reveal meaningful information on the underlying network module structure.

## Introduction

A comprehensive insight into brain processes requires an understanding of information flow between and within structures of the underlying neural system. Commonly, based on non-invasive measuring modalities such as EEG, MEG, and functional MRI (fMRI), this is accomplished by a quantification of directed interactions between recorded time series, where prominent approaches rely on various conceptual principles. Common to all of them is that functional connectivity characteristics are indirectly estimated on the basis of statistical dependencies between measured time series. Due to a limited availability of temporal samples, usually smaller sets of selected or aggregated time series are considered for inferring functional connectivity from estimated statistical characteristics [1,2]. Thus, only little spatially distributed information is ultimately exploited. Yet it is known that brain connectivity phenomena cover many spatially distinct brain regions [3].

An alternative to time series subset pre-selection or aggregation is a reduction of spatial dimensionality by a suitable coordinate transformation such as Principal Component Analysis (PCA), or Independent Component Analysis [4,5]. Their drawback is that interactions between a few principal or independent components are identified, but these interactions cannot be readily transferred back into the original high-dimensional space. This significantly limits the interpretation of functional networks in diseased and healthy states, or in response to behavioral or pharmacological interventions.

Besides approaches for dimension reduction it has also been proposed to circumvent the indeterminacy of parameter estimation equations while keeping the data dimensionality unchanged [6,7]. For instance, this is achieved by means of sparse regression, where a term is additionally inserted into the system of equations, penalizing all non-zero elements of the parameter matrix.

In this work, we propose a novel methodological concept, where spatially high-dimensional data are incorporated into connectivity analysis. These data originate from a (possibly large) system of connected and interacting elements [8] and thus, this system may be considered as a network [9], which represents the functional connectivity structure by linking a set of vertices (recording sites) by edges (interactions). The consideration of spatially high-dimensional data contributes to a much better preservation of the functional attribution of a huge set of network vertices to topological features given by the measuring modality. For functional network identification, the very general predictability principle according to Granger may be used [10]. In neuroscience Granger’s idea has been adapted mostly by using autoregressive models [11,12], which results in linear and fully multivariate methods for inferring functional connectivity. Considering neurophysiological data, the problem of spatially high-resolution data arises in particular in analyses of fMRI data. Here, multivariate approaches such as multivariate autoregressive models (MVAR) suffer from the basic problem that a very high data dimensionality precludes any model fit due to high computational costs or due to the problem of under-determined equations that would have to be solved in the course of model fit.

We propose a general large scale Granger Causality (lsGC) approach, a purely data-driven procedure which involves incorporating a PCA data dimension reduction step into low-dimensional (LD) space, but ultimately attains connectivity patterns in the original high-dimensional (HD) space. This concept is comprised of an orthogonal back projection of LD MVAR model residuals to HD space and using these back-transferred residuals for proper definitions of vertex by vertex interactions.

This preservation of high spatial data dimensionality may result in networks representing functional connectivity consisting of several thousand vertices connected by millions of edges. With methods from network science these large functional connectivity networks can be further processed, and essential information about their underlying connectivity structure can be revealed. In contrast to a segmentation based on vertex activation (voxel time series in the case of fMRI) we are particularly interested in obtaining a functional segmentation of groups of strongly interacting network vertices and in tracing changes in such functionally segmented regions. This functional segmentation can be analyzed naturally within the framework of network module (network community) detection [13,14]. Network modules are a defining topological feature of many network data sets and are given by the clustering of vertices into cohesive groups that represent specialized, functionally indivisible and interacting subnetworks. Typically, these subnetworks are characterized by a larger number of internal interactions and stronger internal interaction patterns that induce more information flow between affiliated vertices as compared to interactions between these subnetworks. In this paper we used directed networks to investigate the causality of interaction patterns by measuring asymmetric propagation of information from source to target vertices.

The paper is organized as follows: Firstly, we describe the generation of a synthetic data set and the real data used in this study. Thereafter, we present the novel lsGCI approach with embedded dimension reduction in detail, followed by an analysis of the effects of dimension reduction on the topology of the resulting lsGCI network using a synthetic network ensemble with known ground truth. We particularly investigate the impact of the edge pattern information loss caused by dimension reduction on the quality and the recoverability of network modules in the resulting lsGCI networks. Then, we give an example for an application of the lsGC approach and use it to obtain high-dimensional networks from resting state fMRI data. Finally, we analyze their functional segmentation given by their module structure and demonstrate that the network modules detected in these functional brain networks are plausible from the physiological perspective.

## Material

### Synthetic ground truth networks and time series generation

To investigate the reliability and suitability of the novel methodology with respect to its effects on the topology and module structure of the resulting networks, we devised a network model to generate synthetic networks with a pre-defined and possibly clear-cut module structure, whose degree of definiteness depends on the chosen parameterization and that should be identifiable by module detection algorithms. In the following, these networks are called ground truth networks.

Each ground truth network consists of *D* ∈ {100,200,…,800} vertices and can be partitioned naturally into non-overlapping modules restricted to consist of ten to fifteen vertices each. The dimensions *D* were chosen to obtain networks that can be still processed with standard GCI methods for the purpose of quantitative comparisons. The number of modules was scaled with the network size, so that for every increase of 100 vertices eight additional modules are gained. The size of each module was chosen randomly, such that the sum of module sizes equals *D*. The edges connecting vertices were placed randomly (uniformly distributed on {10,…,15}) under constraints that define the module structure. Edge patterns were constrained by probabilities for intra-module edges and inter-module edges. All column sums in the resulting adjacency matrices were restricted to be less than or equal to fifteen. In detail, the probability for directed intra-module edges was *p*_int_ = 0.5, whereas the probability for directed inter-module edges *p*_ext_ depends on the network size so that the constraint on the column sums holds true.

To account for outlier vertices that have an above-average number of edges (both, intra-module and inter-module edges) that would violate the column sum constraint, we determined that on average each vertex has three edges to and from vertices of different modules. Consequently, we defined the probability of inter-module edges by *p*_ext_ = 3/(*D* − 15). Moreover, additional conditions on minimum internal and maximum external in- and out-degrees have to hold true, such that the simulated module structure is more clear-cut, while the modules remain connected. That means vertices are limited to maintaining a minimum number of 4 edges both, to and from member vertices of their own module, while their number of interactions with vertices of other modules must not exceed a maximum of 4 edges in each of both directions. The computational costs for the simulations increase strongly with network size *D* as the aforementioned constraints become increasingly harder to satisfy. Finally, we simulated networks up to dimension *D* = 800, for which the computational costs of network generation were still manageable. Based on these settings we generated 100 different instances of ground truth networks for each dimensionality *D*.

The corresponding multivariate time series were realized on the basis of *p*-th order *D*-variate autoregressive (AR) models formally given by

$$Y(n)=\sum_{r=1}^{p}A^{r}\cdot Y(n-r)+E(n),n=p+1,\ldots ,N$$ (1)

with *D*-dimensional state vectors $Y(n)\in R^{D}$ and AR model parameters $A^{r}\in R^{D\times D}$. The model residuals $E\in R^{D\times N}$ are supposed to be zero mean uncorrelated *D*-dimensional random variables.

**Fig 1:**
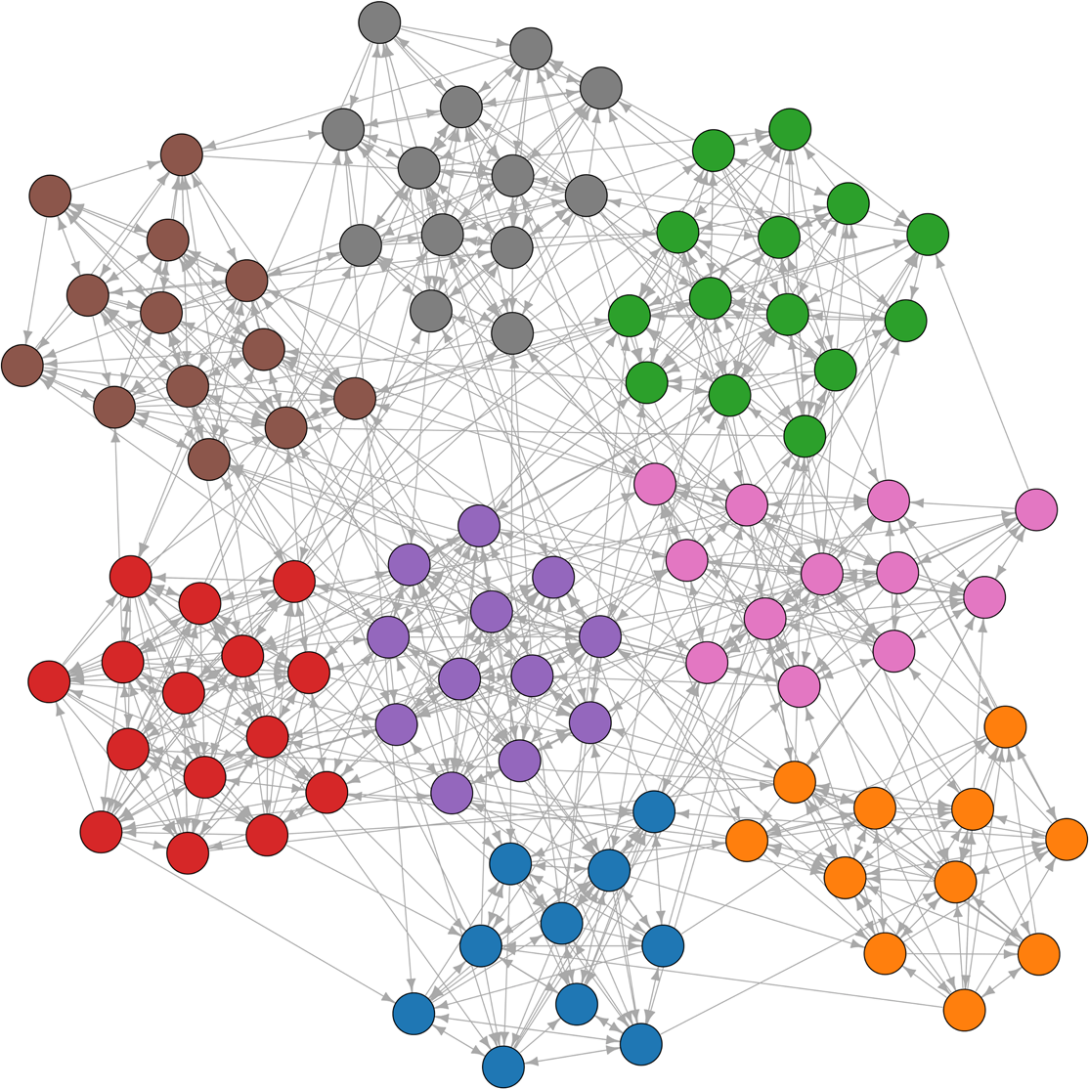
Force directed layout (Fruchterman-Reingold algorithm) of an example ground truth network of dimension *D* = 100.

To obtain time series that possess the previously specified ground truth connectivity patterns, each of these networks was used to generate a *D*-dimensional MVAR process of order *p* = 1. The number of available temporal samples was held constant with *N* = 1000. The effect of *N* was already demonstrated in [15]. The corresponding AR matrices **A**^1^ were defined as follows: if there is no connection from the *j*-th vertex to the *i*-th vertex, the corresponding AR parameter $A_{ij}^{1}$ was set to zero. In the other case, we defined $A_{ij}^{1}=\rho \cdot \frac{0.99}{\eta }$, where *ρ* is uniformly randomly selected from {−1,1} and *η* is the maximum in-degree of all vertices. This scaling ensures the stationarity of the resulting multivariate process. As described above, maximum column sums of all adjacency matrices were restricted to be less than or equal to fifteen, which yields *η* ≤ 15. Thus, the aforementioned constraint provides similar coupling strengths for all network dimensions *D*. The added noise terms E(*n*) were realized by standard normally distributed random numbers. In summary, the generation of synthetic networks was necessarily governed by the autoregressive parameters $A_{ij}^{1}$, which depend directly on *η*. This results in networks with a challenging topology for recovering their module structure. An example of a network structure for *D* = 100 is shown in Fig 1.

### Resting state fMRI data

Five pilot scans used for this study were acquired from HIV positive subjects (four males, one female; mean age: 41 years; age range: 28–53 years) at the Rochester Center for Brain Imaging (Rochester, NY, US) using a 3.0 Tesla Siemens Magnetom TrioTim scanner (Siemens, Erlangen, Germany). The acquisition was approved by the Ethics Committee of the University of Rochester Medical Center (reference number RSRB00042912), and the individuals gave their written consent per protocol. High resolution structural imaging was performed using T1-weighted magnetization-prepared rapid gradient echo sequence (MPRAGE) (TE = 3.44 ms, TR = 2530 ms, isotropic voxel size 1 mm, flip angle = 7). Resting state fMRI scans were acquired using a gradient spin echo sequence (TE = 23 milliseconds, TR = 1650 milliseconds, 96 x 96 (2.5 mm × 2.5 mm) acquisition matrix, flip angle of 84°). Four independent runs were recorded for each subject, where the acquisition of each run lasted 6 minutes with 250 volumes each. A total of 25 slices, each 5 mm thick, was acquired for each volume. During acquisition, subjects were asked to lie still with closed eyes. The first 10 volumes were deleted to allow the signal to reach equilibrium. The volumes were then subjected to slice timing and motion correction as well as brain extraction. Linear detrending was performed by high pass filtering (0.01 Hz). These were then registered to the standard MNI152 template (2 mm isotropic). For subsequent analyses, time series from ventricles were masked out using the standard ventricle mask based on the MNI152 template available in FSL [16]. All preprocessing steps were carried out using FEAT (FMRI Expert Analysis Tool), which is part of FSL and its respective subroutines.

## Methods

### Large scale Granger Causality

#### Granger Causality

The general concept of Granger Causality (GC) is based on the core idea that the cause precedes its effect. One possible approach to quantify this notion refers to the principle of predictability: a variable Y_*i*_ Granger-causes another variable Y_*j*_ of the same multivariate process if the knowledge of Y_*i*_'s past improves the the forecast of Y_*j*_ [10]. In this work, the GC concept is realized by means of multivariate autoregressive models according to Eq (1), whereby the resulting model residual terms are used to define an appropriate measure of predictability. For this purpose, a data matrix $y=(y(1),\ldots ,y(N))\in R^{D\times N}$ containing *D* measured time series with *N* temporal samples is approximated by a *D*-dimensional, *p*-th order MVAR process according to (1). The fitted model is then given by the difference between the original data **y** and the resulting model residuals $e\in R^{D\times N}$

$$\hat{y}=y-e.$$ (2)

One possible way to estimate the model parameters is to rearrange Eq (1) such that the model residuals can be minimized using the method of multivariate least squares [17].

Within this framework, a Granger Causality Index (GCI) can be determined in terms of the model prediction errors: Y_*i*_ is said to be Granger-causal to Y_*j*_ if the prediction error of Y_*j*_ is increased by excluding the past of Y_*i*_. More precisely, let $y^{i-}\in R^{(D-1)\times N}$ be the reduced data matrix where the *i*-th row of **y** is eliminated, and let **e**^*i*−^ the resulting residuals of the corresponding (*D* − 1)-variate MVAR model fit. Then, the MVAR-based GCI from Y_*i*_ to Y_*j*_ is quantified on the basis of the estimated covariance **Σ** = Cov(**e**) of the full process and the estimated covariance **Σ**^*i*−^ = Cov(**e**^*i*−^) of the reduced process by

$$\gamma_{i\to j}=ln\left(\frac{\Sigma_{j}^{i-}}{\Sigma_{j}}\right),$$ (3)

where $\Sigma_{j}^{i-}$ and Σ_*j*_ denote the diagonal entries of **Σ**^*i*−^ and **Σ** associated to Y_*j*_ respectively.

#### Principal component analysis

Principal component analysis is a widely used procedure for dimension reduction and feature extraction. The basic idea is to linearly transform input data into a new set of variables, such that the major proportion of data variation is explained by a few derived components. More precisely, by means of PCA, the input data $y\in R^{D\times N}$ are orthogonally mapped onto another *D*-dimensional space such that a few resulting uncorrelated components account for the major proportion of variance in the original data. This linear transformation can be represented by the equation

$$x=W^{D}\cdot y$$ (4)

with the PCA mixing matrix $W^{D}\in R^{D\times D}$ and the matrix containing the *D* principal components $x\in R^{D\times N}$. It can be demonstrated that the base vectors of this space are provided by the eigenvectors of **y**; the corresponding eigenvalues then contain the proportion of explained variance within each eigenvector direction [18]. Let *λ*_1_,…,*λ*_*D*_ denote the diagonal entries of the covariance matrix of **x**. Then the proportion of explained variance given by the first *C* principal components amounts to $\sum_{c=1}^{C}\lambda_{c}/\sum_{c=1}^{D}\lambda_{c}$. In practice, it is not always necessary to retain all derived components as the proportion of explained variance that is provided by the last components is frequently quite low (the components are usually sorted in descended order of their variance explanation). Accordingly, only a few decorrelated components are necessary for the explanation of a major proportion of variance. This has the advantage that analyses of the high-dimensional data can be performed on the basis of the low-dimensional derived principal components whilst retaining the major part of information provided by the high-dimensional data.

#### Large scale Granger Causality

A drawback of the MVAR approach is that the model parameter estimation is not feasible for very high-dimensional data. The reason is that for a non-singular estimation equation, the condition

N−p≥D⋅p (5)

has to be fulfilled. When the number of sampling points *N* is by far below the number of network vertices *D*, condition (5) cannot be fulfilled and it is thus impossible to attain a least square solution for the MVAR model.

In the following we describe how PCA and the MVAR-based GCI approach can be beneficially combined to overcome this problem whilst maintaining the full spatial resolution. In a first stage, PCA is used as a preliminary data reduction step in order to enable the MVAR model estimation in the low-dimensional (LD) space under a maximum of variance explanation. Let $x\in R^{C\times N}$ be the matrix containing only the first *C* PCA-transformed vectors of the original data matrix **y** according to Eq (4)

x=W⋅y, (6)

with the truncated mixing matrix $W=(W_{1},\ldots ,W_{C})\in R^{C\times D}$. The benefit of this is that the problem of singular estimation equations can be circumvented: as long as the number of retained components *C* and the MVAR model order *p* are chosen sufficiently low, the MVAR model estimation equation for **x** instead of **y** is not singular and the LD MVAR model can be fitted. According to (5), this is the case if *N* − *p* ≥ *C* · *p* is fulfilled. Assuming this condition is satisfied, let $\hat{x}$ be the least square MVAR approximation of **x**. The error variance of the LD model residuals $\hat{x}-x$ can then be used to calculate the GCI *λ*_*i*→*j*_ between the principal components *i* and *j*, 1 ≤ *i* ≠ *j* ≤ *C*. However, this does not reflect the causal relationship between HD network vertices *i* and *j*, 1 ≤ *i* ≠ *j* ≤ *D*. Thus, instead of this LD approach yielding connectivity patterns between *C* principal components, it would be desirable to have a tool that enables a HD vertex-by-vertex quantification of directed interactions.

The large scale Granger Causality index is a measure that is supposed to counter this issue by offering a possibility for the quantification of connectivity between HD vertices [15,19]. The main idea is to initially utilize the above described PCA-based data projection from HD space into LD space (4) in order to enable the least square estimation of a *C*-variate MVAR model. Then, instead of using the LD model residuals $\hat{x}-x\in R^{C\times N}$ for the calculation of GCI, a HD error term is defined that enables the vertex-by-vertex definition of an adequate GCI at the large scale.

To obtain such an error term, $\hat{x}$ is linearly transferred back into HD space by minimizing the sums of least squares. This procedure leads to the back-projected model $W^{+}\cdot \hat{x}$, where **W**^+^ denotes the pseudo inverse of the PCA matrix **W** in Eq (6). Then, the HD residuals amount to the difference between the original data and the back-transferred MVAR model time series $\hat{x}$

$$\hat{e}(n)=\hat{y}(n)-W^{+}\hat{x}(n)\in R^{D}$$ (7)

The LD variable, where the influence of the HD signal y_*i*_ is excluded, can be assessed in two ways: either by canceling the *i*-th row of the original data matrix **y** and performing a *separate* PCA calculation for each of the *D* cancellation steps, or by performing *one* PCA and canceling the *i*-th row of the original data together with the *i*-th column of the PCA mixing matrix **W**. It has been demonstrated in a simulation study that the latter procedure outperforms the first one [15]. Hence the analyses in this study will be limited to the second approach, which will be described in the following. Let **x**^*i*−^ be the LD variable where the influence of the HD signal y_*i*_ is eliminated by

$$x^{i-}=W^{i-}\cdot y^{i-},$$ (8)

where $W^{i-}\in R^{C\times (D-1)}$ is the matrix containing all columns but the *i*-th of the PCA mixing matrix **W** and $y^{i-}\in R^{(D-1)\times N}$ denotes the data matrix where the *i*-th observed time series of **y** is eliminated. The reduced HD residuals are defined as

$${\hat{e}}^{i-}(n)=y(n)-{\left(W^{i-}\right)}^{+}{\hat{x}}^{i-}(n).$$ (9)

Then the HD prediction error covariances $\tilde{\Sigma }=Cov(\hat{e})$ and ${\tilde{\Sigma }}^{i-}=Cov({\hat{e}}^{i-})$ are used for the calculation of the large scale Granger Causality index from y_*i*_ to y_*j*_, 1 ≤ *i* ≠ *j* ≤ *D*. It is defined as

$${\tilde{\gamma }}_{i\to j}=ln\left(\frac{{\tilde{\Sigma }}_{j}^{i-}}{{\tilde{\Sigma }}_{j}}\right).$$ (10)

**Fig 2:**
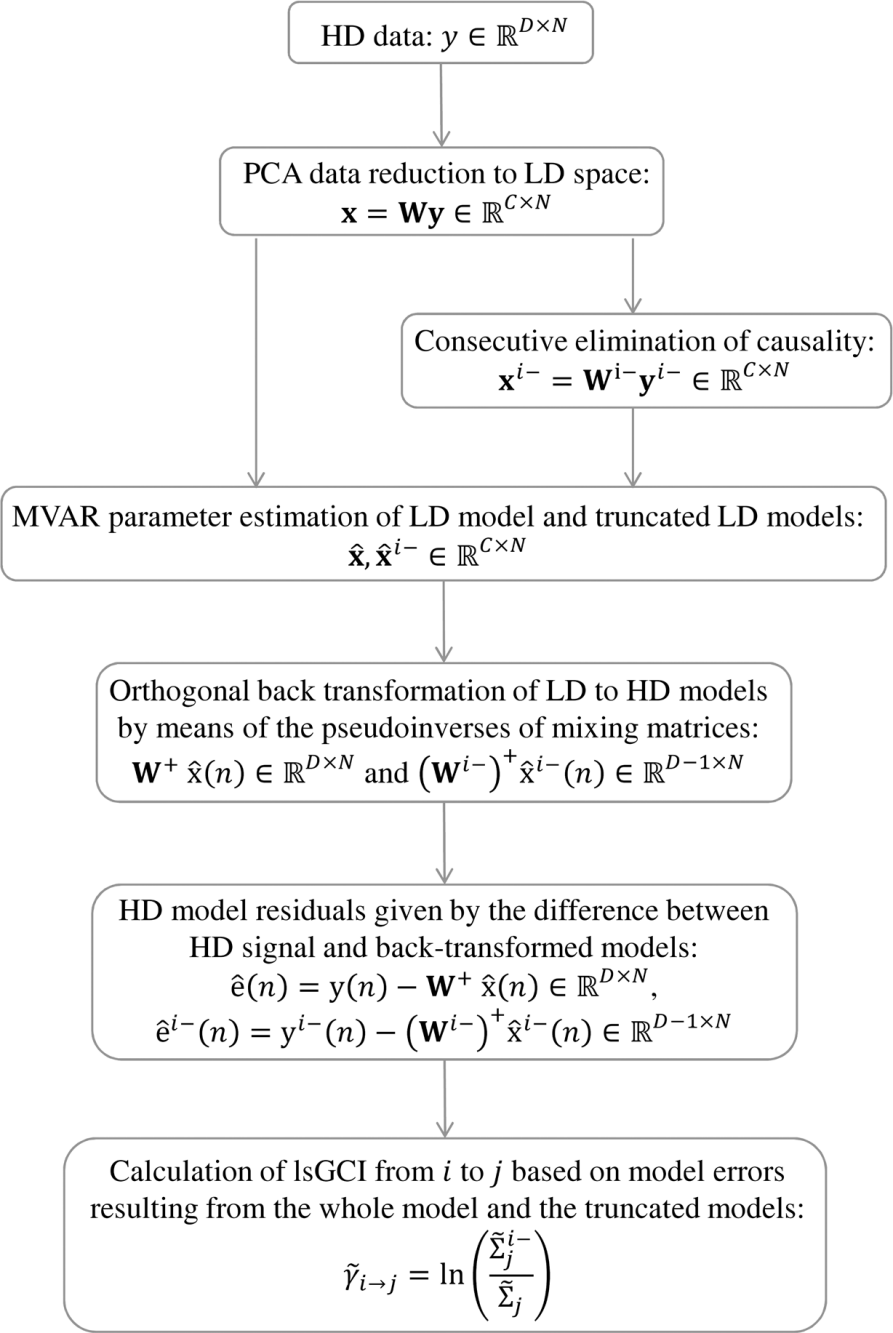
Overview of successive processing steps in the lsGCI calculation.

Fig 2 provides a summary of the whole procedure showing all consecutive steps that have to be performed in the course of lsGCI calculation: first, the HD signal **y** is transformed to LD space by conventional PCA and the LD MVAR model is estimated. Then, successively, the influence from each signal y_*i*_ is removed by eliminating the *i*-th column of **W** and the *i*-th row of **y** and then the MVAR model ${\hat{x}}^{i-}$ for the resulting LD data is estimated. As a next step, all models are transferred back into HD space via left multiplication of the pseudo inverse of the original mixing matrix **W**^+^ and the pseudo inverse of the altered mixing matrix (**W**^*i*−^)^+^, respectively. Finally, HD residuals are obtained by taking the difference between original signal and back-transferred estimated models, which are finally used for the calculation of the lsGCI values (10).

### Dichotomization of large scale GCI networks

Up to now there has been no generally accepted criterion for thresholding functional connectivity measures to define non-complete binary directed networks. There are in general two principle approaches: statistical significance tests [20–22] and deterministic thresholding procedures [22–24]. Statistical methods are burdened by the arbitrary definition of the type I error and the way alpha-adjustments for multiple comparisons are performed, if any. In contrast, heuristic thresholds are chosen somewhat arbitrarily or attempt to fix arbitrary values of basic network characteristics, such as vertex degrees or edge density, which yield a common cutoff level for all network edges. Among other pitfalls heuristic thresholds are possibly introducing several kinds of biases to the characteristics of the resulting network topology, which are hard to compensate [22].

For the fMRI lsGCI networks we used the multiple threshold strategy to yield binary networks according to the literature [23]. Multiple thresholds are used to define and consequently remove subliminal (i.e. weak or spurious) interactions from the weighted complete networks whose edge weights are given by the connectivity measure. The resulting networks are then dichotomized by assigning all remaining edges the weight 1. We used 90%, 95% and 98% percentile values of the edge weight distribution defined in advance as threshold levels. Relatively high thresholds were chosen to preferentially obtain networks with a reduced edge density. Furthermore, the range of thresholds was constrained by the connectedness of the resulting networks, i.e. the generated binary networks were not allowed to fragment into separate connected components. That is, single isolated vertices or single subnetworks, which would cause problems for some module detection algorithms and which would not represent a good model of (functional) brain connectivity from the neurophysiological point of view. For the analysis of the effects of the lsGCI procedure on network module recoverability using synthetic ground truth networks, we have chosen network-specific thresholds that yielded binary networks with maximum similarity to the ground truth networks. Formally, the thresholds were determined according to a maximum Cohen’s kappa [25].

### Algorithms for module structure identification

For our analysis we used various algorithms for identifying module structure. A module structure identification algorithm reveals a network partition (also called a clustering) of vertices into modules (subsets of similar or related vertices that are also called clusters or communities) by assigning densely interconnected vertices the same module affiliation. To obtain such a classification of vertices, the algorithms typically rely only on structural information contained in the edge patterns of the network. Since the module detection algorithms use different strategies to exploit and interpret structural information inherent to the network data, an identified partition is not necessarily unique, thus different partitions of similar quality and equal legitimacy might exist. If multiple results are returned by an algorithm (e.g. if the search strategy entails uncovering hierarchies of network partitions) we always select the partition with the highest modularity.

We used the “leading eigenvector” algorithms of Newman [26] (undirected networks) and Leicht & Newman [27] (directed networks), the “Louvain” algorithm of Blondel et al. [28] (directed and undirected networks), the “fast greedy” algorithm of Clauset et al. [29] (undirected networks), the “Walktrap” algorithm [30] (undirected networks), the “Infomap”' [31] (directed networks) and the Potts spin glass based module detection [32] (undirected networks). We are concerned with directed networks, while some of these methods were specifically developed for undirected networks. In general, edge directions can encode potentially useful functional information that should not be discarded. While it might seem theoretically appealing to take the directed nature of networks into account to identify modules, the effect of edge orientation on module detection accuracy is not immediately clear and might not be generalizable from one network data set to another, as an edge in any direction indicates a potential similarity of vertices by virtue of their interaction. Following this reasoning, we applied algorithms designed for undirected networks on symmetrized versions of our directed adjacency matrices in which any directed edge is replaced by an undirected one.

### Module structure quality characteristics

To evaluate and quantify the effect of the lsGCI procedure with varying degrees of dimension reduction on the recoverability and quality of obtained network modules we used several network characteristics for comparing the lsGCI-derived network module structure with the one in their respective synthetic ground truth networks, e.g. modularity, mutual information, variation of information, split-join distance, partition edit distance or the performance index, among others. We give a concise description of these measures together with appropriate references in the captions of the figures presented in the Supporting Information of this article. Here we describe only the ratio of correctly classified vertices, the Rand index and the coverage measure, which we refer to in the main article.

#### Ratio of correctly classified vertices

The ratio of correctly classified vertices is obtained by comparing the network partition yielded by a particular module detection algorithm with another benchmark classification of vertices that is either assumed to be correct or is correct by construction. It measures the “goodness” of a partition simply by counting misclassified vertices. To compute this percentage, module labels of vertices in each lsGCI network have to be matched to the ones identified in each ground truth network. The matching is performed by defining a cost matrix based on the Jaccard distance for all pairs of modules and solving the resulting assignment problem. This approach causes problems when the number of uncovered modules is different, e.g. one module in the ground truth network was found to be separated into two modules in the corresponding lsGCI network. In this case one of the two subsets of the original module cannot be matched and consequently the ratio of correctly classified vertices will be greatly reduced by virtue of this split, even though a large fractions of the same vertex pairs are still clustered together. This problem is handled more robustly by the Rand index.

#### Rand index

The Rand index [33–35] compares two different network partitions *P*_*A*_ and *P*_*B*_ of the same vertex set on the basis of counting and comparing classifications of pairs of vertices in both partitions. Thereby, it does not make use of topological information, i.e. adjacency information of the network. The Rand index is given by $R({P_{A},P}_{B})=\frac{N_{11}+N_{00}}{N_{11}+N_{00}+N_{01}+N_{10}}$, where *N*_11_ represents the number of vertex pairs that are assigned to the same module (vertices that are clustered together) in both *P*_*A*_ and *P*_*B*_, while *N*_00_ is the number of vertex pairs that are assigned to different modules in both *P*_*A*_ and *P*_*B*_. There are two more types of classified vertex pairs: They represent disagreement, that is, pairs of vertices that are assigned to different modules in *P*_*A*_ but are placed in the same module in *P*_*B*_ (*N*_01_) and pairs of vertices where the situation is the opposite (*N*_10_).

#### Coverage

Coverage denotes the ratio of the number of intra-module edges by the total number of edges. The motivation behind it is the following: in an ideal module structure, e.g. if a network is fully fragmented into isolated connected components with no inter-module edges linking vertices of different connected components, there would be little ambiguity with respect to the (non-hierarchical) module structure. In this case all edges are intra-module edges and the value of coverage is one. Thus, coverage measures the goodness of the obtained network partition into modules in dependence of the quality of the network's inherent module structure.

## Results

### Synthetic data

In the case of classical GCI (i.e. 100% explained variance), it is known that an increase of sample size *N* enhances the identifiability of connectivity patterns due to an improved MVAR estimation.

The comparison between original GCI and lsGCI was carried out by means of ROC (Receiver Operating Characteristic) curve analysis, which basically corresponds to the systematic thresholding concept as described above. In the case of artificial data, the underlying ground truth was known and therefore the ROC status variable could be defined by means of the entries in the adjacency matrix, i.e. presence (status = positive) or absence (status = negative) of an directed edge. An aspect that has to be considered in the course of the lsGCI procedure is the amount of HD data variance that can be explained by LD data after the dimension reduction step (6). This quantity is directly linked to the number of retained components *C*: the higher *C*, the higher the explained variance. In order to determine the influence of this parameter setting on lsGCI results, we performed the analysis with various *C* (variance explanations), whereby *C* = *D* represents 100% variance explanation and thus the classical GCI.

**Fig 3:**
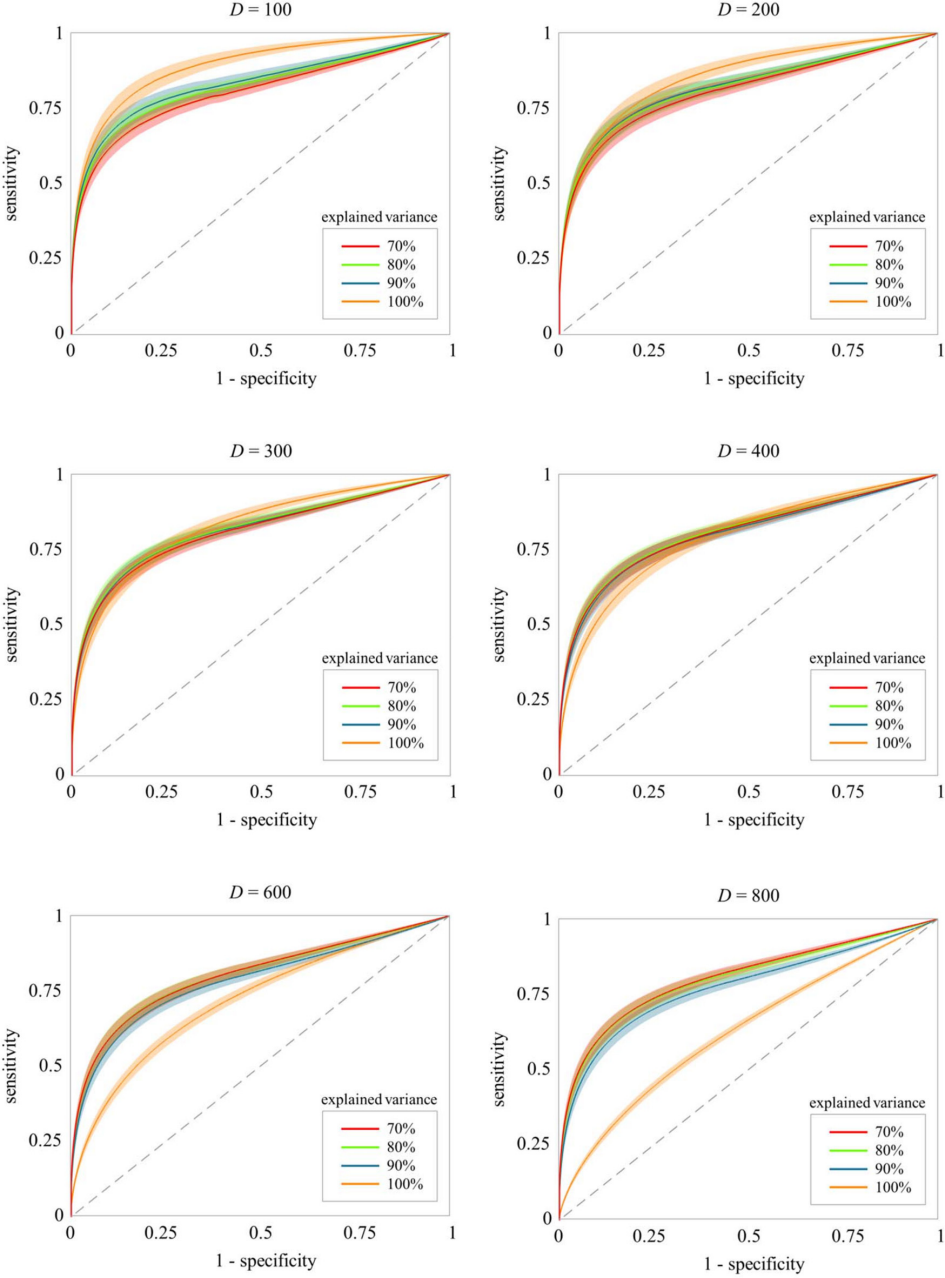
The lsGCI results for synthetic data: mean ROC curves (100 realizations) with standard deviation bands for *D* = 100,200,300,400,600,800, *N* = 1000, and various variance explanations. A variance explanation of 100% corresponds to the classical GCI, whereas all other shown variance explanations correspond to the lsGCI.

**Fig 4:**
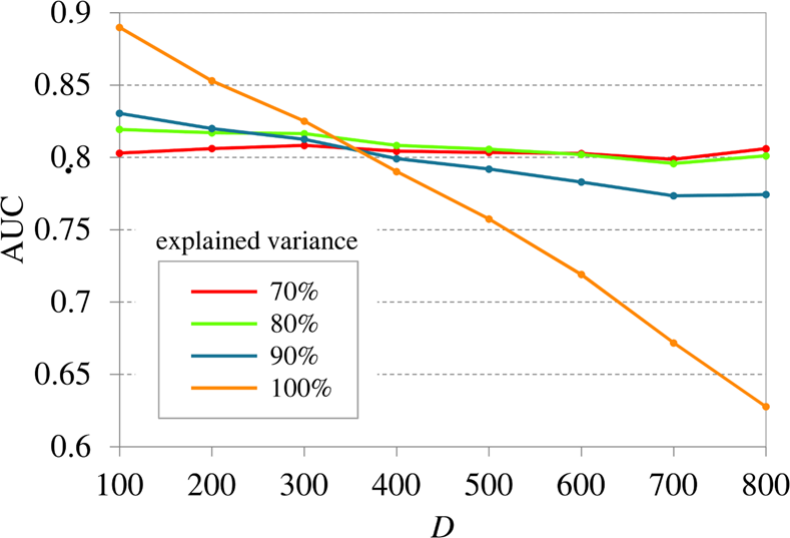
The lsGCI results for synthetic data: mean area under ROC curves (100 realizations) for several dimensions *D* = 100,200,300,400,600,800, *N* = 1000, and various variance explanations. The maximum standard error of all estimations equals 0.0023, which is too small to be plotted. A variance explanation of 100% corresponds to the classical GCI, whereas all other shown variance explanations correspond to the lsGCI.

Fig 3 depicts the results of ROC curve analysis for various dimensions *D* and several variance explanations. All curves relate to averaged results with respect to the realizations of 100 ground truth networks. The corresponding standard deviations are depicted as surrounding bands. The (ls)GCI ROC curves indicate that a higher amount of explained variance leads to a better performance for smaller dimensions *D* ≤ 300. Yet, all lsGCIs achieved reasonable results despite the loss of information due to the PCA dimension reduction. Here as expected, the classical GCI without embedded dimension reduction outperforms the lsGCI approach, where the difference between GCI and lsGCI decreases when *D* increases. In particular, the performance decrease turns out stronger for the classical GCI in comparison to the lsGCI. Consistently, beginning with *D* ≥ 400 the situation is changing and the embedded dimension reduction shows a positive effect since the stronger performance decrease of the GCI continues while the lsGCI performance marginally decreases with increasing *D*. Fig 4 compactly demonstrates this behavior on the basis of the area under the ROC curve.

**Fig 5:**
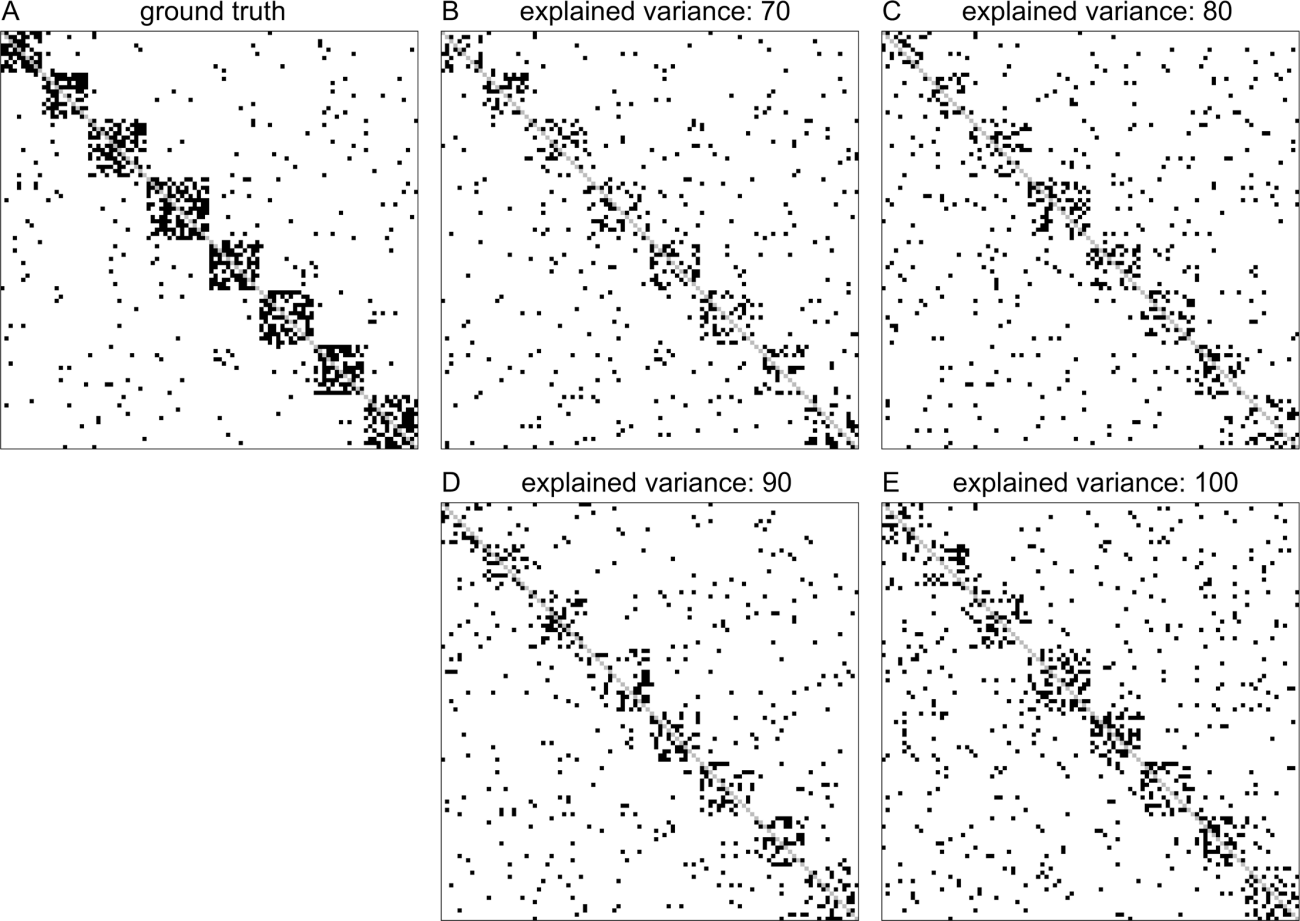
Plots of example adjacency matrices of the ground truth network and corresponding lsGCI networks with different degrees of dimension reduction (*D* = 100, *N* = 1000). (A) ground truth network; (B-D) lsGCI network with variance explanations from 70%-90%; (E) GCI network.

In addition to ROC curve analysis we considered the effect of different degrees of dimension reduction on edge pattern alterations and the recoverability of network modules in dichotomized lsGCI networks. To give a visual impression of these effects, the adjacency matrices of an exemplary ground truth network with *D* = 100 and its corresponding lsGCI networks are depicted in Fig 5. A force-directed network layout of an example ground truth network is depicted in Fig 1.

**Fig 6:**
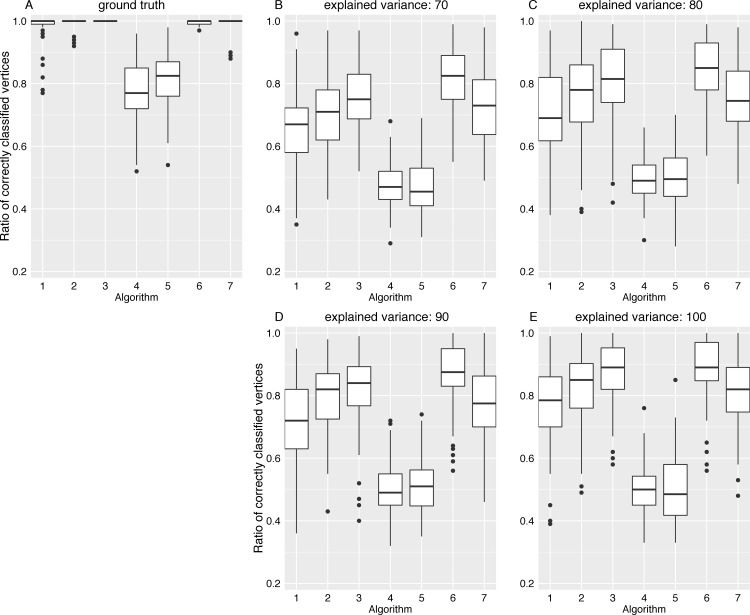
The ratio of correctly classified vertices of the ground truth network and lsGCI networks (*D* = 100, *N* = 1000). The following algorithms for network module identification were used: “leading eigenvector” (1), “Louvain” directed (2),“Walktrap” (3), “fast greedy” (4), “leading eigenvector” (5), “Potts spin glass” (6), “Louvain” undirected (7). (A) ground truth network; (B-D) lsGCI network with variance explanations from 70%-90%; (E) GCI network.

**Fig 7:**
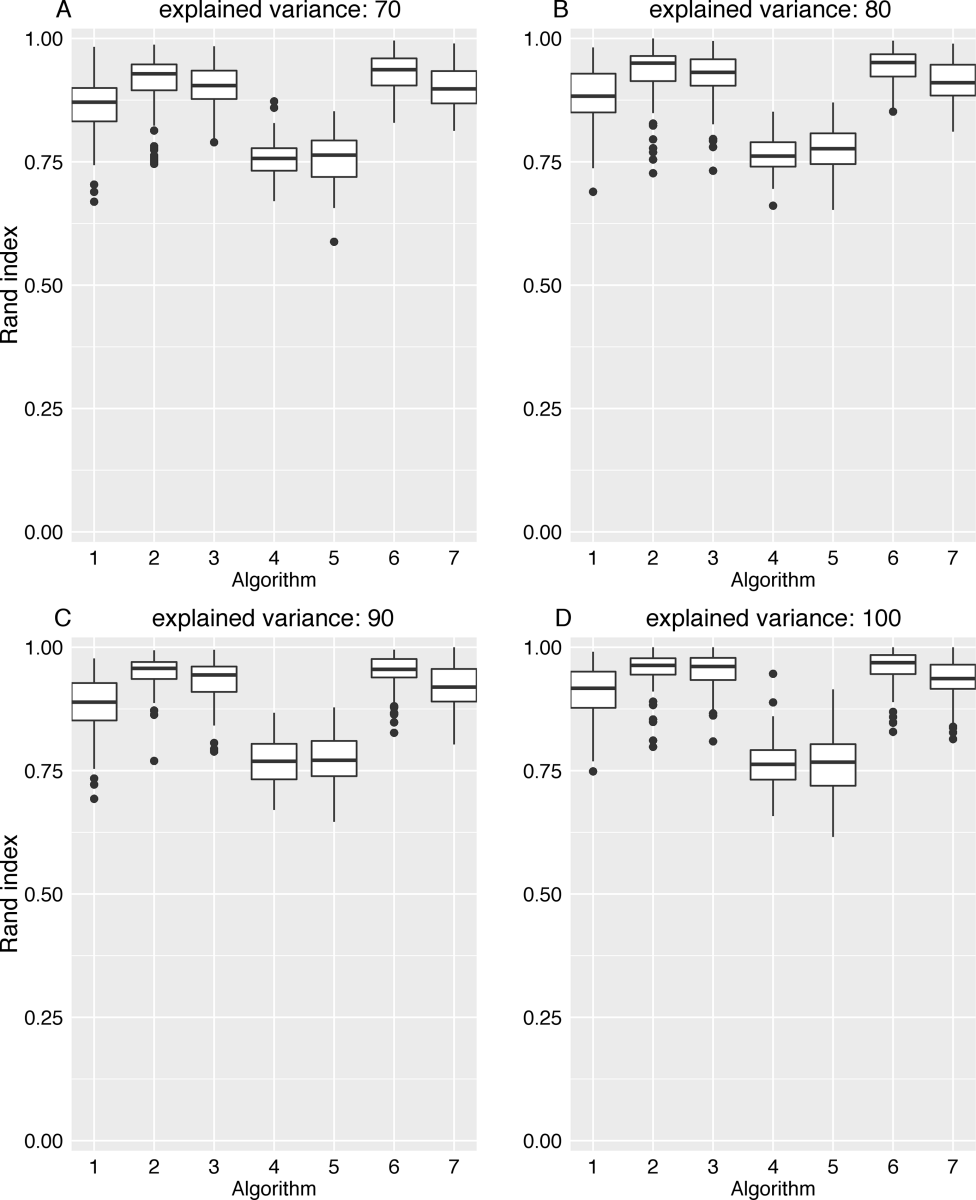
Rand index for the ground truth network and lsGCI networks (*D* = 100, *N* = 1000). The following algorithms for network module identification were used: “leading eigenvector” (1), “Louvain” directed (2), “Walktrap” (3), “fast greedy” (4), “leading eigenvector” (5), “Potts spin glass” (6), “Louvain” undirected (7). (A) ground truth network; (B-D) lsGCI network with variance explanations from 70%-90%; (E) GCI network.

**Fig 8:**
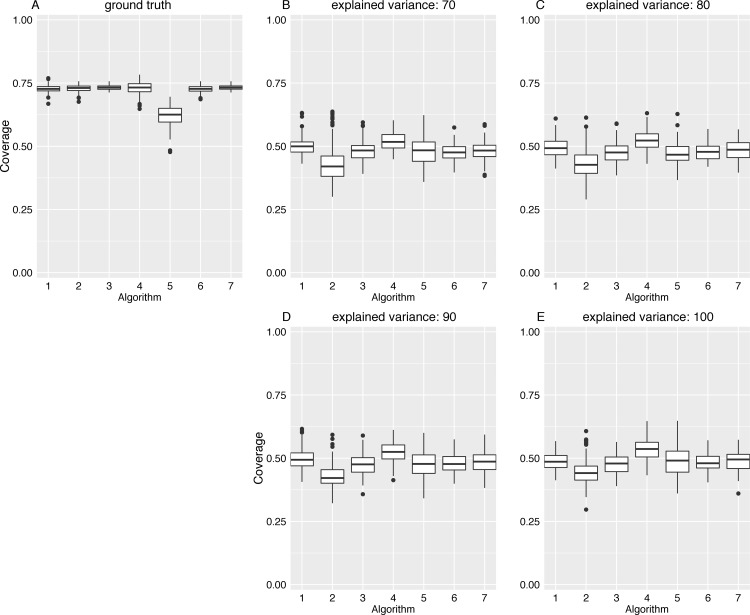
Coverage values for the ground truth network and lsGCI networks (*D* = 100, *N* = 1000). The following algorithms for network module identification were used: “leading eigenvector” (1), “Louvain” directed (2), “Walktrap” (3), “fast greedy” (4), “leading eigenvector” (5), “Potts spin glass” (6), “Louvain” undirected (7). (A) ground truth network; (B-D) lsGCI network with variance explanations from 70%-90%; (E) GCI network.

The adjacency matrix image in Fig 5(A) shows the connections in the ground truth network, while the adjacency matrix in Fig 5(E) shows the edge pattern alterations caused by the classical GCI approach. In this figure, results for an increasing variance explanation are shown from panel (B) to panel (E). As expected, depending on the degree of dimension reduction, inter-module edge patterns were considerably altered. Although intra-module module edge patterns were thinned out, the module structure of each lsGCI network is still apparent by visual inspection. To quantify the effects of dimension reduction we analyzed different topological and information theoretic properties of module structures identified in the synthetic ground truth networks and the corresponding lsGCI network ensembles with different degrees of dimension reduction. The various network characteristics we used cover many different aspects of network module structure and give a coherent picture of topological alterations provided by lsGCI matrices. Despite the information loss inflicted by the dimension reduction step and its associated edge pattern alterations, we found that the module recoverability as well as the quality of the identified network partitions was still good in the lsGCI networks (especially for the range of explained variance that is of practical relevance), as compared to their respective ground truth networks, even when considerably reduced. For this analysis, the results regarding the percentage of correctly classified vertices, the Rand index, and the coverage are presented in Fig 6, Fig 7 and Fig 8, respectively. We refer to the supporting information for results of all module structure characteristics.

Notably, for all considered module identification algorithms, we found that the percentage of correctly classified vertices in all lsGCI networks was high, even though slightly reduced in comparison to the ground truth networks. Depending on the module detection algorithm used and the amount of explained variance, the median percentage of correctly classified vertices for lsGCI networks was between 47% and 87.5%, whereas for the the ground truth networks it was in the range between 77% and 100%. For some of the module detection algorithms the identified module affiliations of vertices in the lsGCI networks were similar to the ones obtained from the ground truth networks. The results for the ratio of correctly classified vertices are presented in Fig 6. As mentioned in section Methods, the ratio of correctly classified vertices potentially yields a distorted picture of the module detection results if the number of identified modules does not coincide with the number of modules in the ground truth network. Therefore, we also show the results for the Rand index, which measures the similarity of the module structure detected in the ground truth networks with the one in the lsGCI network: The boxplots in Fig 7 show that a large fraction of vertex pairs are clustered together or separated into different clusters in an identical fashion in the ground truth networks and lsGCI networks. Depending on the module detection algorithm and the amount of variance explanation the median Rand index is in the range between 0.76 and 0.96, which is close to its maximum.

The partition distance (variation of information) [36], mutual information [36], “split-join” distance [37] and the Rand index [33–35] are measures that depend only on the vertex affiliations of two network partitions that are compared with each other. In our results their values were still in line with the recoverability of vertex module affiliations and showed that the lsGCI networks were similar to their ground truth networks from the perspective of module identification. However, when comparing these information theoretic and cluster similarity measures, the negative influence of increasing degrees of dimension reduction (Fig 5B–5E) appeared more pronounced (increased interquartile ranges) compared to some of the measures which exploit topological information in addition to vertex affiliations (see below).

To further contrast the generated module structure of the ground truth networks with the one of the lsGCI networks we consider network characteristics that take into account the module affiliations of vertices as well as features of network topology. Such characteristics yield a particularly accurate picture of the degree to which the lsGCI network structure was impaired. We found that the values of network characteristics like modularity [14,27], performance measure and coverage [13] were noticeably reduced in comparison to the ground truth networks. The (partition) edit distance of intra-module edges [38,39] was relatively high, reflecting the changes of intra-module edges patterns in the lsGCI networks. As an example, in Fig 8 we show the effect of dimension reduction on coverage values obtained for all considered samples of lsGCI networks. We observed a considerable decline in coverage values as compared to the respective ground truth networks, whose coverage values had substantial magnitudes (they were between 0.63 and 0.73, depending on the module identification algorithm). The negative influence of increasing degrees of dimension reduction (seen in the panels from (E) to (B)) turned out to be small. Also, the considered module identification algorithms yielded mostly consistent results. The same general behavior was observed for the other above mentioned characteristics, which confirmed our initial findings from inspecting the adjacency matrix plots. The boxplots for all analyzed network characteristics can be found in the supporting information.

### Resting state fMRI data

**Fig 9:**
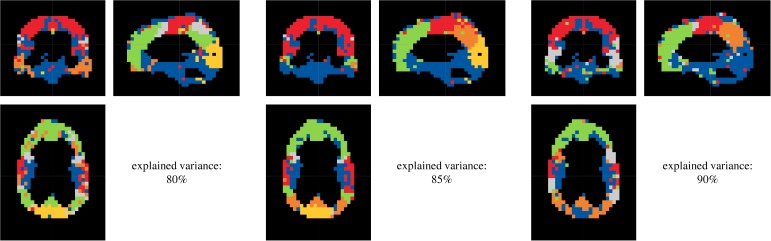
Results of the resting state fMRI functional segmentation for different variance explanations (subject E, 95% edge weight distribution percentile, “Louvain” directed). Each color represents one identified module.

**Fig 10:**
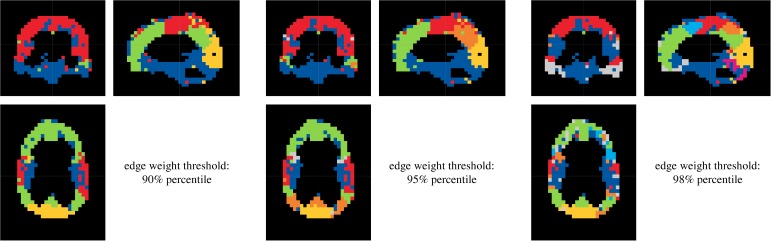
Results of the resting state fMRI functional segmentation for different edge weight distribution percentiles (subject E, 85% variance explanation, “Louvain” directed). Each color represents one identified module.

**Fig 11:**
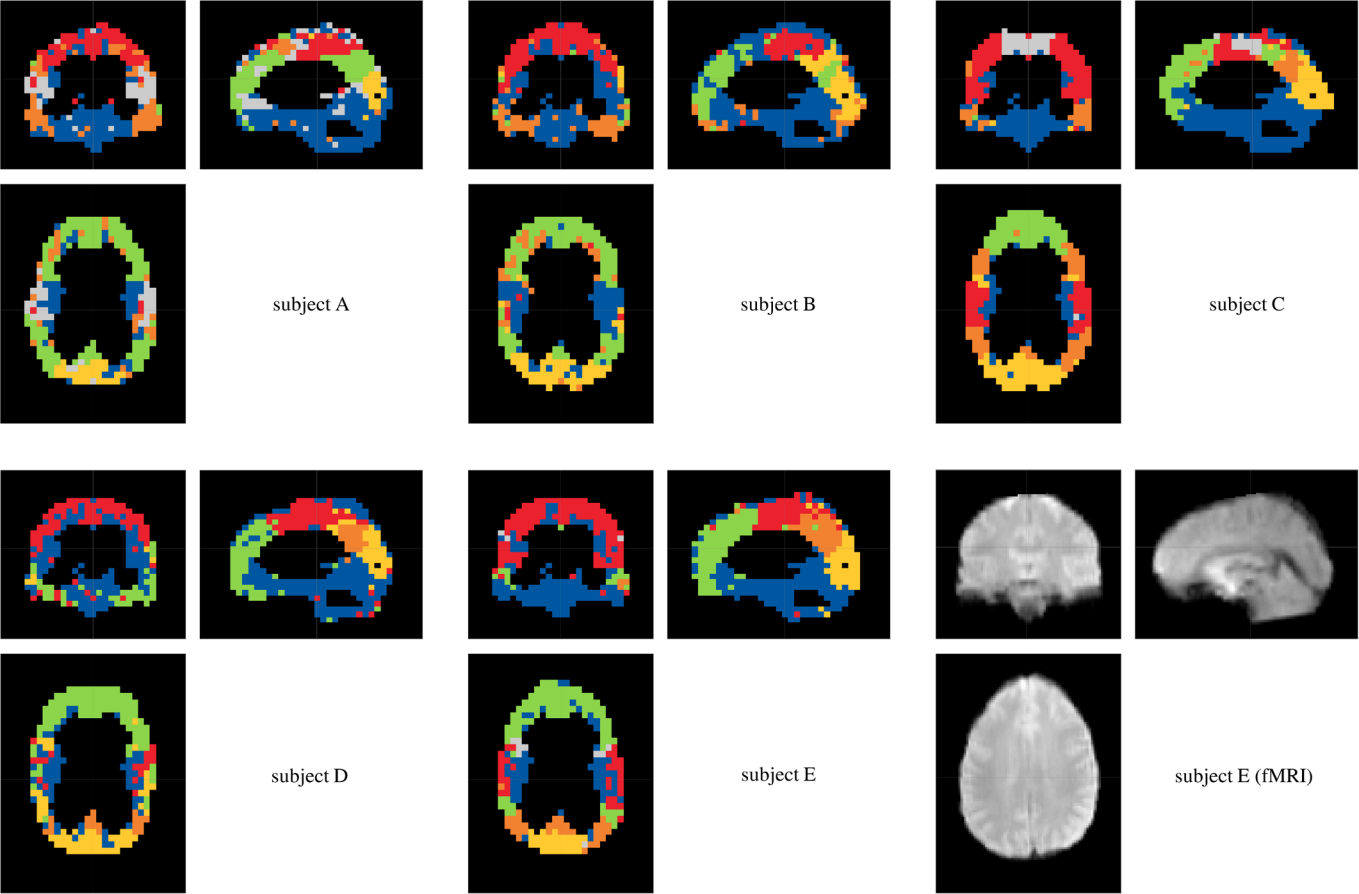
Results of the resting state fMRI functional segmentation for different subjects (85% variance explanation, 95% edge weight distribution percentile, “Louvain” directed). Each color represents one (subject-individual) identified module. As reference, one fMRI volume is shown bottom right (registered to the standard MNI152 template).

Connectivity analysis of clinical data was limited to every third voxel, in sagittal, frontal and transverse direction, still resulting in *D* = 5723 to *D* = 6007 voxel time series to be processed. The aim of this procedure was to avoid excessively speckled module patterns, enabling a physiologically reasonable partitioning. Using Akaike's information criterion and needing a sufficient fit between parametric AR and empirical FFT spectra, the MVAR model order was set to *p* = 5. The amount of variance explanation was variably chosen between 80% and 90%. Furthermore, thresholds for deterministic dichotomization of lsGCI values varied between 90% and 98% edge weight distribution percentiles. Similarly to the results of the synthetic data, it turned out that the portion of explained variance had only a minor influence on assigned module affiliations (Fig 9), while the effect of dichotomizing the networks using different global thresholds had a more pronounced effect on the perceived quality and definiteness of the identified module structure (Fig 10). Also, the quality of results varied according to which module detection algorithm was applied. Frequently, the “leading eigenvector” algorithms of Newman and Leicht and Newman, the “Louvain” algorithm of Blondel *et*.*al* for directed and undirected networks and the “Potts spin glass” algorithm yielded clearly outlined network modules for our data. These algorithms differently use structural information to identify a vertex partition into modules, yet they all yield plausible and relatively similar network partitions. To give an example for the entire group, a projection of module affiliations identified by the Louvain algorithm for directed networks (for an 95% percentile edge weight threshold) is depicted in Fig 11.

The coloring signifies identified module membership of voxels. Clearly, the affiliation of spatially distributed voxels to modules did not occur unsystematically. Rather, the voxel modules closely followed the conventional classification of the lobes of the brain. Indeed, it is possible to classify the lobes of the brain for all persons analyzed. Especially, this classification is representable for subjects E and C. In comparison to it, the lobes are less clear for subject B. All five persons analyzed show a demarcation by voxels of the area of the precentral gyrus (primary motor cortex) and postcentral gyrus (primary sensory cortex) to the frontal lobe and parietal lobe, respectively. This module is particularly indicated for subjects E and C (red module). Large differences for the classification of the lobes according to the different adjustments of variance explanation and dichotomization threshold are not visible. The distribution of voxels with the same color was almost symmetric in a comparison of the left and right hemispheres of the brain and nearly follows the lobe classification of the brain. Yet generally, the adjustments seem to be coarser for lower variance explanations than for higher. Finally, these differences are marginal. In summary it can be stated, that the resulting module structure (derived based on functional connectivity patterns) is very alike to anatomical structures for all five persons analyzed.

It would be desirable that modules with similar topological properties, composition or location are color-matched. However, this objective cannot be universally achieved, e.g. because a module of one subject may be disaggregated into several modules of another subject. As a consequence, related modules of different subjects might be colored by different colors, which complicates group analyses. We frequently observed such situations in our data set, too.

## Discussion

The investigation of functional networks within high-dimensional data is commonly associated with computational or analytical problems due to the high data volume. This issue is frequently addressed by restricting connectivity analyses to specific regions of interest or to low-dimensional components that account for the major portion of data variance.

As an alternative, we have proposed the large-scale Granger Causality approach that takes advantage of a PCA-based dimension reduction whilst allowing a high-dimensional quantification of functional connectivity. Within this framework, we explicitly exploited the linearity of MVAR models such that a straightforward embedding of a linear dimension reduction into the whole time series modeling procedure is adequate. Theoretically it is conceivable to transfer the proposed idea to alternative time series models and dimension reduction procedures. However, from the current perspective, the restriction to linear models offers the most practical and reliable approach. However, the linearization caused by the underlying MVAR model induces that non-linear phenomena may not be inferred, which is one of the frequently mentioned qualified points of critique. John Geweke appositely called this type of Granger Causality “measure of linear feedback”. In order to tackle a higher data dimensionality we have focused on the concept of (linear) Granger Causality because of the possibility of a fully multivariate analysis, the capability of unveiling directed interaction patterns and its potential to be generalized. In that sense, the linearization may be considered as concession on the way to the analysis of high-dimensional multivariate time series. In contrast, model-based approaches in low-dimensional spaces have been proven to be very effective because of concrete connectivity assumptions. Assumptions regarding the temporal behavior of single network vertices as well as physiological constraints can be integrated, and non-linear effects may be modeled as well [40–43].

The embedded dimension reduction step introduces one additional parameter, namely the variance explanation (or number of retained components), which is necessary for subsequent LD model fits. Selecting an optimal degree of dimension reduction is still an open question. From our perspective, it does not seem to be appropriate to explain as much variance as technically possible. From the practical point of view, variance explanations of around 80% yielded consistent and reasonable results for a multitude of data sets, in particular for real fMRI data. Moreover, in this range we observed a remarkable stability in the sense that the number of retained components does not influence the detected network module affiliations as much as one might expect.

From the practical point of view, an appropriate choice of *C* can be supported by an empirical approach. Firstly due to technical reasons, there is an upper limit for *C* because the inequality *N* − *p* ≥ *C* · *p* must be fulfilled. This inequality also defines the maximum possible amount of variance explanation. Regarding the lower limit, we found that variance explanations less than 65% yielded meaningful results only in rare cases. Within these limits, an exhaustive analysis of different variance explanations seems to be reasonable since it turned out that there is usually a broader range with very stable analysis results. Finally, *C* should be chosen from this range.

In the course of lsGCI calculation there are several aspects that might influence the quality of the results. These can be divided into two groups: data properties and analysis settings. Data properties (such as time series length *N* and number of network vertices *D*) are in practice naturally determined by the available data, while the analysis settings (such as model order and number of retained PCA components) can be chosen by the user. Based on our experience, the influence of these parameters on the results can be summarized as follows: the performance of the lsGCI approach is enhanced with an increasing number of temporal samples and is slightly decreased with an increasing number of network vertices (Fig 3 and Fig 4). We furthermore observed that, if the model order is chosen too high, the results get worse due to the increasing number of MVAR model parameters that have to be estimated. The behavior for a model order that is chosen too low could not be investigated in this simulation study as the true model order was set to *p* = 1. However, it is clear that an underestimated model order necessarily leads to poor results because the process characteristics are not properly reflected by the MVAR model [44,45]. Our investigations have shown that the influence of the parameter *C* is depending on data properties such as the number of available temporal samples *N* and the number of network vertices *D*. In the case of a high *N* in relation to *D*, the effect is as expected: the performance of lsGCI is enhanced with an increasing proportion of explained variance (Fig 3). If applicable, the classical GCI should be preferred opposite to lsGCI in this case. When the relationship between *N* and *D* shifts in favor of *D* the lsGCI approach with a reduced dimensionality leads to a better performance (Fig 3 and Fig 4) compared to GCI. Moreover, the performance of lsGCI approach shows only a relatively small dependence on *D*. This effect has not been clarified yet in its entirety, but an explanation might be that the imbalance between *D* and *N* leads to poor GCI results because a too small number of temporal samples in combination with a high number of network vertices leads to a high variability of estimated model residuals. Consequently, the PCA dimension reduction enhances the quality of MVAR estimation in spite of the loss of a small amount of explained variance.

To validate our new approach with benchmark data, we generated a large sample of ground truth networks with pre-defined module structures. The dichotomization of lsGCI networks was performed by a systematic thresholding procedure. As expected, a diminished portion of explained data variance results in an impaired recovery of the true network structure. This applies for both, false positive and false negative rates. For appropriate percentages of explained variance we found that intra-module edges and inter-module edges are altered by the dimension reduction step in a balanced way, so that information about the original module structure contained in a ground truth network is preserved to a large degree. This enables module identification algorithms to uncover a very high percentage of the true module affiliations of vertices in the lsGCI networks. It can be seen in our synthetic network data for which we found a minor impact of the lsGCI procedure on the recoverability of network modules despite profound alterations of edge patterns.

In addition to the degree of dimension reduction given by the number of retained components and the edge weight dichotomization strategy, the quality of the identified module structure depends strongly on the module identification algorithm used. There is a wealth of module identification algorithms described in the literature, each of them having different computational complexity, using different algorithmic strategies and exploiting different features of network topology to uncover network partitions. Therefore, it is a priori not unequivocally clear which module identification algorithm would yield the best results in an acceptable runtime. We applied many different module identification algorithms and found that in general network modules could be reliably identified for the lsGCI networks based on the synthetic ground truth data. The difference between the results of the module identification algorithms was bigger for the lsGCI networks based on high-dimensional fMRI recordings. Here some of the applied algorithms (“Walktrap”, “Infomap”, “fast greedy”) identified either only one module or only an implausible module structure consisting of a large number of small modules that were split and scattered randomly across their network.

High spatial resolution in combination with a comparably low number of volumes, as for example in the case of fMRI data, leads to a high number of time series that are linearly dependent. This property can be beneficially utilized by means of a PCA dimension reduction step as very few principal components account for the major percentage of explained variance. Due to the lack of an available ground truth for real world data, evaluation of the results is mainly based on plausibility arguments. In the case of our analyzed data, this plausibility was provided by a similarity between identified network modules and anatomical lobe classification.

In summary, without a dimension reduction step it is not possible to obtain functional connectivity networks of similar high dimensionality. The application of the proposed method to clinical fMRI data now offers the opportunity to assess the feasibility of the long-term perspective of a full-brain functional connectivity analysis.

## Supporting Information

S1 Fig Modularity values for the ground truth network and lsGCI networks. By means of accounting for the magnitude of local edge densities, modularity measures how clear-cut a network partition is [Fortunato, 2010; Leicht and Newman, 2008; Newman, 2012; Newman and Girvan, 2004]. It is defined as the difference of the fraction of intra-module edges and the expected fraction of such edges in a suitable random network. Modularity for directed networks takes into account “surprising” edges given the degree information of their tail and head vertices, e.g. edges that fall between pairs of vertices where the tail-vertex has small out-degree and the head vertex has small in-degree. The following algorithms for network module identification were used: “leading eigenvector” (1), “Louvain” directed (2), “Walktrap” (3), “fast greedy” (4), “leading eigenvector” (5) “Potts spin glass” (6), “Louvain” undirected (7). (A) ground truth network; (B-D) lsGCI network with variance explanations from 70%-90%; (E) GCI network.
(TIFF)

S2 Fig Mutual information of the ground truth network and lsGCI networks. The reduction of uncertainty of vertex assignments in one partition due to knowledge about the other partition is given by the mutual information [Meilă, 2007]. The following algorithms for network module identification were used: “leading eigenvector” (1), “Louvain” directed (2), “Walktrap” (3), “fast greedy” (4), “leading eigenvector” (5), “Potts spin glass” (6), “Louvain” undirected (7). (A) ground truth network; (B-D) lsGCI network with variance explanations from 70%-90%; (E) GCI network.
(TIFF)

S3 Fig Partition edit distance between the ground truth network and lsGCI networks. This measure computes for each pair of corresponding modules in both partitions the Levenshtein edit distance [Dasgupta et al., 2006; Levenshtein, 1966] of intra-module edges, which is the cost for their optimal alignment. For it, the adjacency matrix for each module is vectorized and typecasted to a string in the alphabet {0,1}. The Levenshtein distance is the minimum number of insertions, deletions and substitutions to make both strings equal. Single edit distances for each pair of corresponding modules are added up to yield the partition edit distance. The following algorithms for network module identification were used: “leading eigenvector” (1), “Louvain” directed (2), “Walktrap” (3), “fast greedy” (4), “leading eigenvector” (5), “Potts spin glass” (6), “Louvain” undirected (7). (A) ground truth network; (B-D) lsGCI network with variance explanations from 70%-90%; (E) GCI network.
(TIFF)

S4 Fig Performance measure for the ground truth network and lsGCI networks. For a network partition uncovered by a module detection algorithm, the fraction of correctly “interpreted” vertex pairs with regard to the network adjacency information is called performance [Fortunato, 2010]. It takes into account the vertex pairs that are assigned the same module and that interact via an edge and those vertex pairs where both vertices are classified to belong to different modules that are not connected by an edge. In other words, the performance measure penalizes edges that are ignored by a given network partition (when both end-vertices are assigned to different modules) and it penalizes edges implied by the network partition that are not present in the network (vertices with the same module affiliation should ideally be connected by an edge). The following algorithms for network module identification were used: “leading eigenvector” (1), “Louvain” directed (2), “Walktrap” (3), “fast greedy” (4), “leading eigenvector” (5) “Potts spin glass” (6), “Louvain” undirected (7). (A) ground truth network; (B-D) lsGCI network with variance explanations from 70%-90%; (E) GCI network.
(TIFF)

S5 Fig Adjusted Rand index for the ground truth network and lsGCI networks. In practice the Rand index does not necessarily range over the entire [0,1] interval and instead often concentrates in a small interval close to 1. Therefore, it might be adjusted for chance assignment of modules [Hubert and Arabie, 1985]. The following algorithms for network module identification were used: “leading eigenvector” (1), “Louvain” directed (2), “Walktrap” (3), “fast greedy” (4), “leading eigenvector” (5), “Potts spin glass” (6), “Louvain” undirected (7). (A) ground truth network; (B-D) lsGCI network with variance explanations from 70%-90%; (E) GCI network.
(TIFF)

S6 Fig Split-join distance between the ground truth network and lsGCI networks. The split-join distance [Dongen, 2000] measures the extent to which two partitions are subpartitions of each other by means of accounting for their module overlap. The following algorithms for network module identification were used: “leading eigenvector” (1), “Louvain” directed (2), “Walktrap” (3), “fast greedy” (4), “leading eigenvector” (5), “Potts spin glass” (6), “Louvain” undirected (7). (A) ground truth network; (B-D) lsGCI network with variance explanations from 70%-90%; (E) GCI network.
(TIFF)

S7 Fig Variation of information of the ground truth network and lsGCI networks. The variation of information [Meilă, 2007] compares two network partitions by measuring the change in their information (using entropy and mutual information) when one partition is converted to the other one. It is a metric on the space of network partitions and can consequently be used to calculate the distance of two partitions of the same network data. This measure does not depend on topological information of the input network as it relies only on module affiliations of vertices. The following algorithms for network module identification were used: “leading eigenvector” (1) “Louvain” directed (2) “Walktrap” (3), “fast greedy” (4), “leading eigenvector” (5), “Potts spin glass” (6), “Louvain” undirected (7). (A) ground truth network; (B-D) lsGCI network with variance explanations from 70%-90%; (E) GCI network.
(TIFF)

S1 File Supporting references. (PDF)
