# Supplementary figures and images for: A Multivariate Granger Causality Concept towards Full Brain Functional Connectivity
Source: PLoS One. 2016 Apr 11;11(4):e0153105. doi: 10.1371/journal.pone.0153105 (PMC4827851; doi:10.1371/journal.pone.0153105)

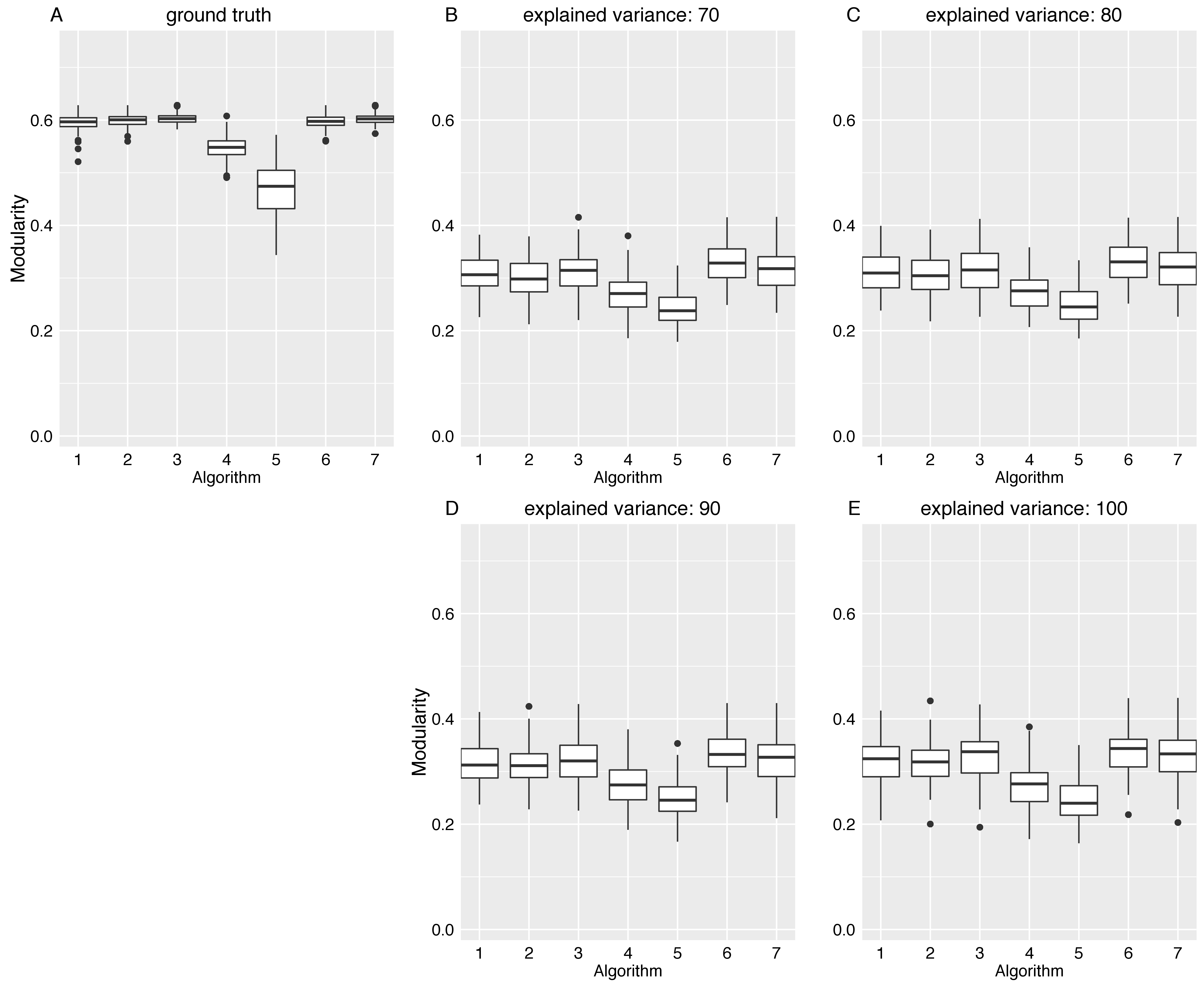

Supplement: S1 Fig — By means of accounting for the magnitude of local edge densities, modularity measures how clear-cut a network partition is [Fortunato, 2010; Leicht and Newman, 2008; Newman, 2012; Newman and Girvan, 2004]. It is defined as the difference of the fraction of intra-module edges and the expected fraction of such edges in a suitable random network. Modularity for directed networks takes into account “surprising” edges given the degree information of their tail and head vertices, e.g. edges that fall between pairs of vertices where the tail-vertex has small out-degree and the head vertex has small in-degree. The following algorithms for network module identification were used: “leading eigenvector” (1), “Louvain” directed (2), “Walktrap” (3), “fast greedy” (4), “leading eigenvector” (5) “Potts spin glass” (6), “Louvain” undirected (7). (A) ground truth network; (B-D) lsGCI network with variance explanations from 70%-90%; (E) GCI network. (TIFF) [file pone.0153105.s001.tiff]

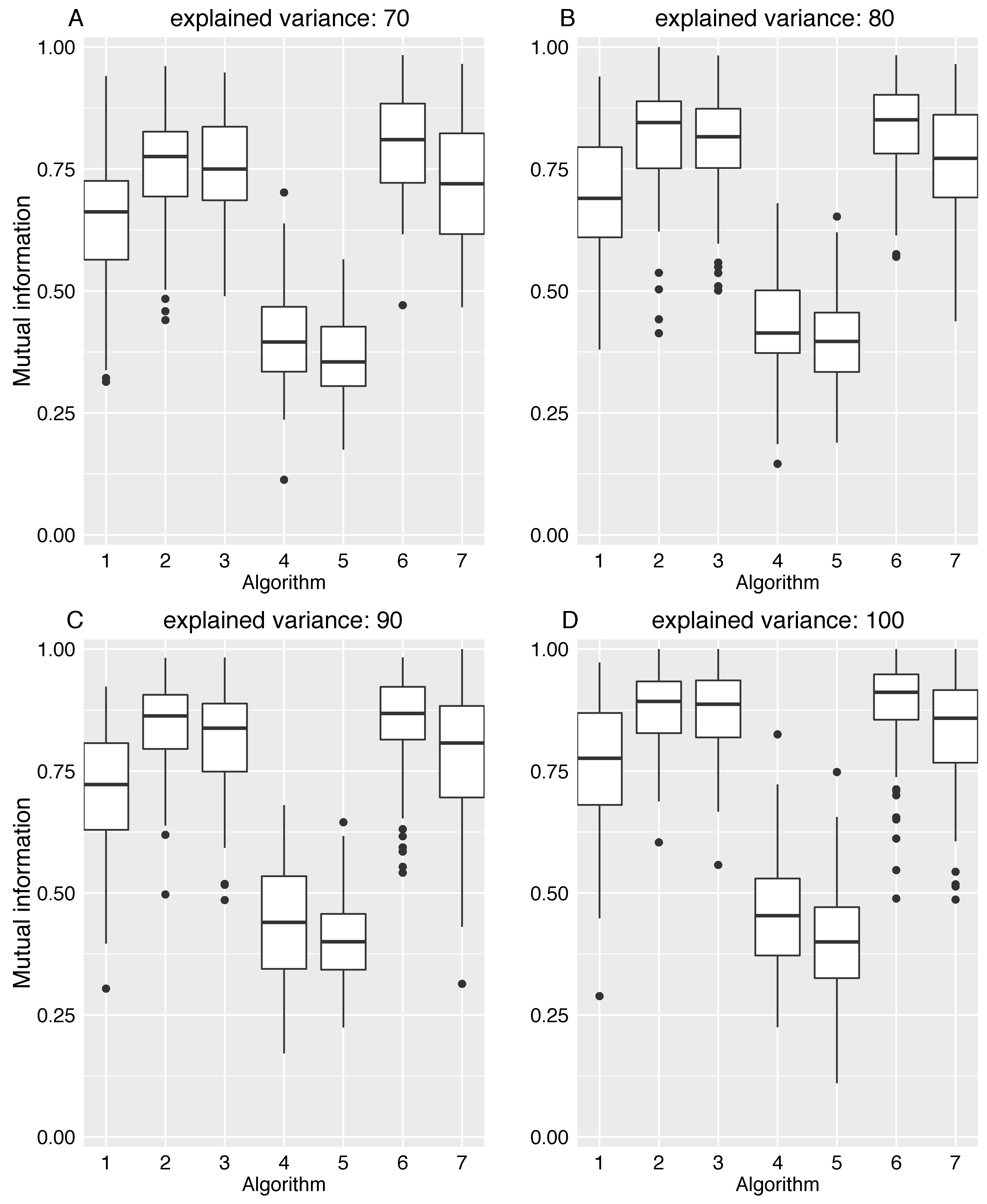

Supplement: S2 Fig — The reduction of uncertainty of vertex assignments in one partition due to knowledge about the other partition is given by the mutual information [Meilă, 2007]. The following algorithms for network module identification were used: “leading eigenvector” (1), “Louvain” directed (2), “Walktrap” (3), “fast greedy” (4), “leading eigenvector” (5), “Potts spin glass” (6), “Louvain” undirected (7). (A) ground truth network; (B-D) lsGCI network with variance explanations from 70%-90%; (E) GCI network. (TIFF) [file pone.0153105.s002.tiff]

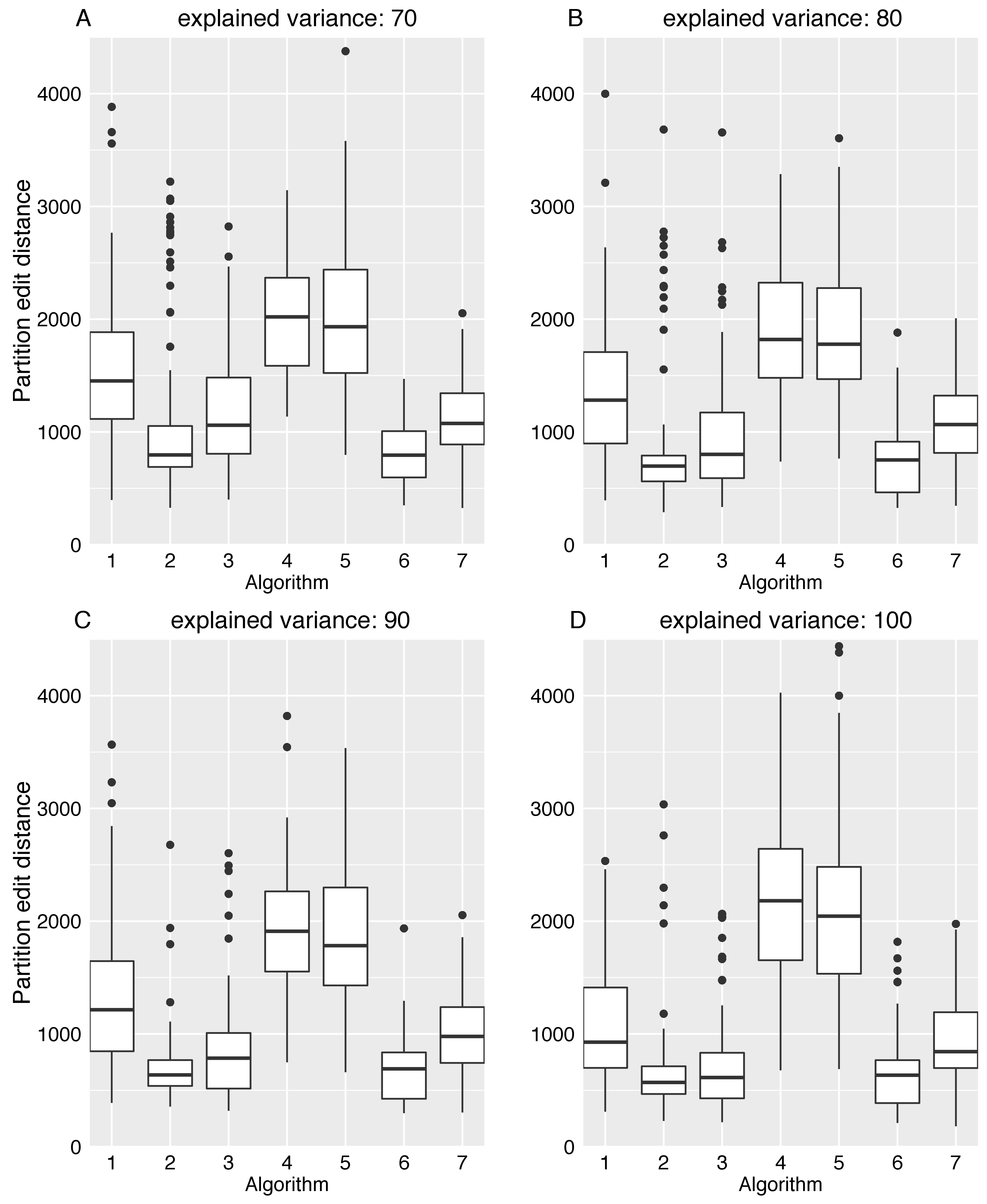

Supplement: S3 Fig — This measure computes for each pair of corresponding modules in both partitions the Levenshtein edit distance [Dasgupta et al., 2006; Levenshtein, 1966] of intra-module edges, which is the cost for their optimal alignment. For it, the adjacency matrix for each module is vectorized and typecasted to a string in the alphabet {0,1}. The Levenshtein distance is the minimum number of insertions, deletions and substitutions to make both strings equal. Single edit distances for each pair of corresponding modules are added up to yield the partition edit distance. The following algorithms for network module identification were used: “leading eigenvector” (1), “Louvain” directed (2), “Walktrap” (3), “fast greedy” (4), “leading eigenvector” (5), “Potts spin glass” (6), “Louvain” undirected (7). (A) ground truth network; (B-D) lsGCI network with variance explanations from 70%-90%; (E) GCI network. (TIFF) [file pone.0153105.s003.tiff]

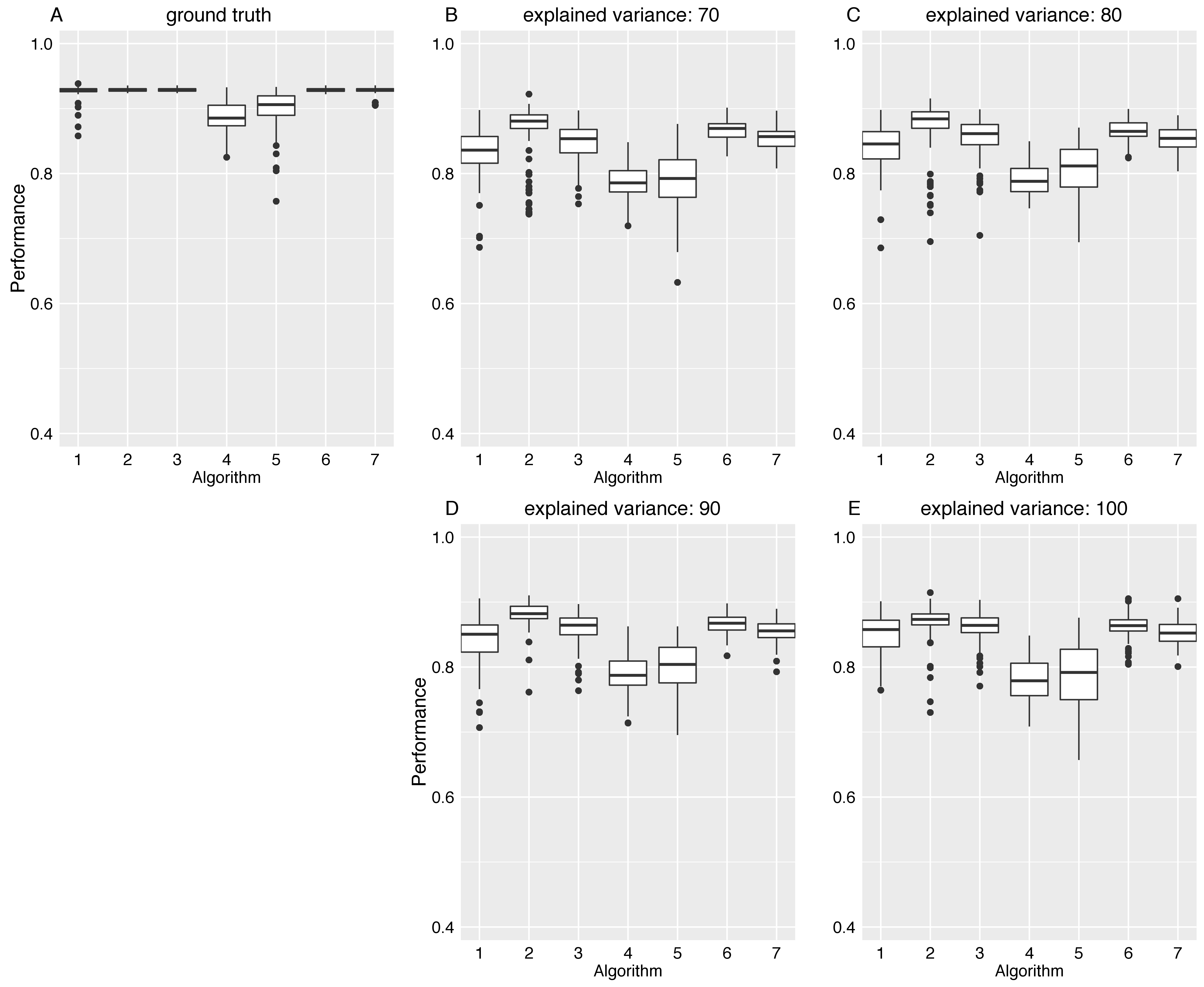

Supplement: S4 Fig — For a network partition uncovered by a module detection algorithm, the fraction of correctly “interpreted” vertex pairs with regard to the network adjacency information is called performance [Fortunato, 2010]. It takes into account the vertex pairs that are assigned the same module and that interact via an edge and those vertex pairs where both vertices are classified to belong to different modules that are not connected by an edge. In other words, the performance measure penalizes edges that are ignored by a given network partition (when both end-vertices are assigned to different modules) and it penalizes edges implied by the network partition that are not present in the network (vertices with the same module affiliation should ideally be connected by an edge). The following algorithms for network module identification were used: “leading eigenvector” (1), “Louvain” directed (2), “Walktrap” (3), “fast greedy” (4), “leading eigenvector” (5) “Potts spin glass” (6), “Louvain” undirected (7). (A) ground truth network; (B-D) lsGCI network with variance explanations from 70%-90%; (E) GCI network. (TIFF) [file pone.0153105.s004.tiff]

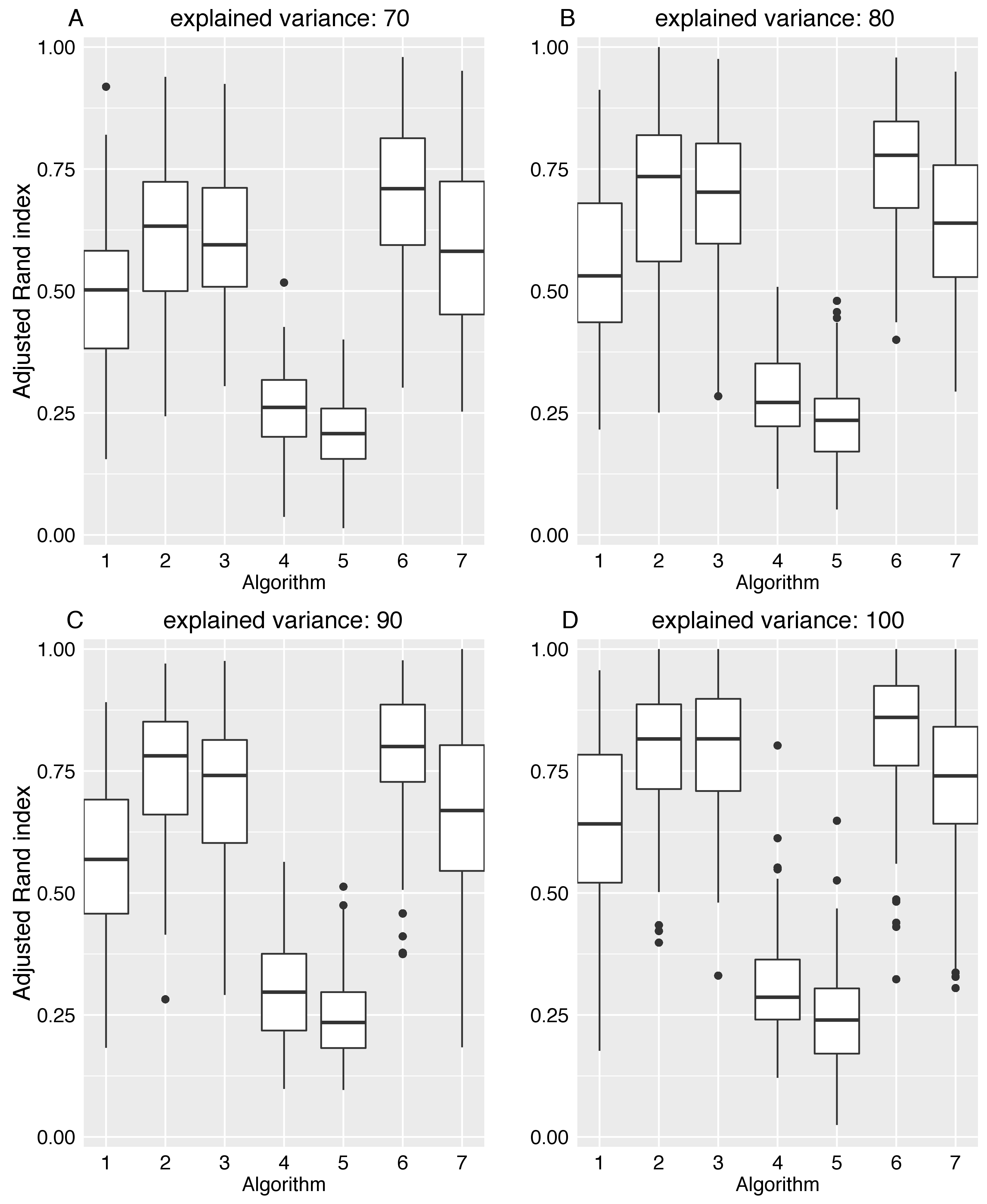

Supplement: S5 Fig — In practice the Rand index does not necessarily range over the entire [0,1] interval and instead often concentrates in a small interval close to 1. Therefore, it might be adjusted for chance assignment of modules [Hubert and Arabie, 1985]. The following algorithms for network module identification were used: “leading eigenvector” (1), “Louvain” directed (2), “Walktrap” (3), “fast greedy” (4), “leading eigenvector” (5), “Potts spin glass” (6), “Louvain” undirected (7). (A) ground truth network; (B-D) lsGCI network with variance explanations from 70%-90%; (E) GCI network. (TIFF) [file pone.0153105.s005.tiff]

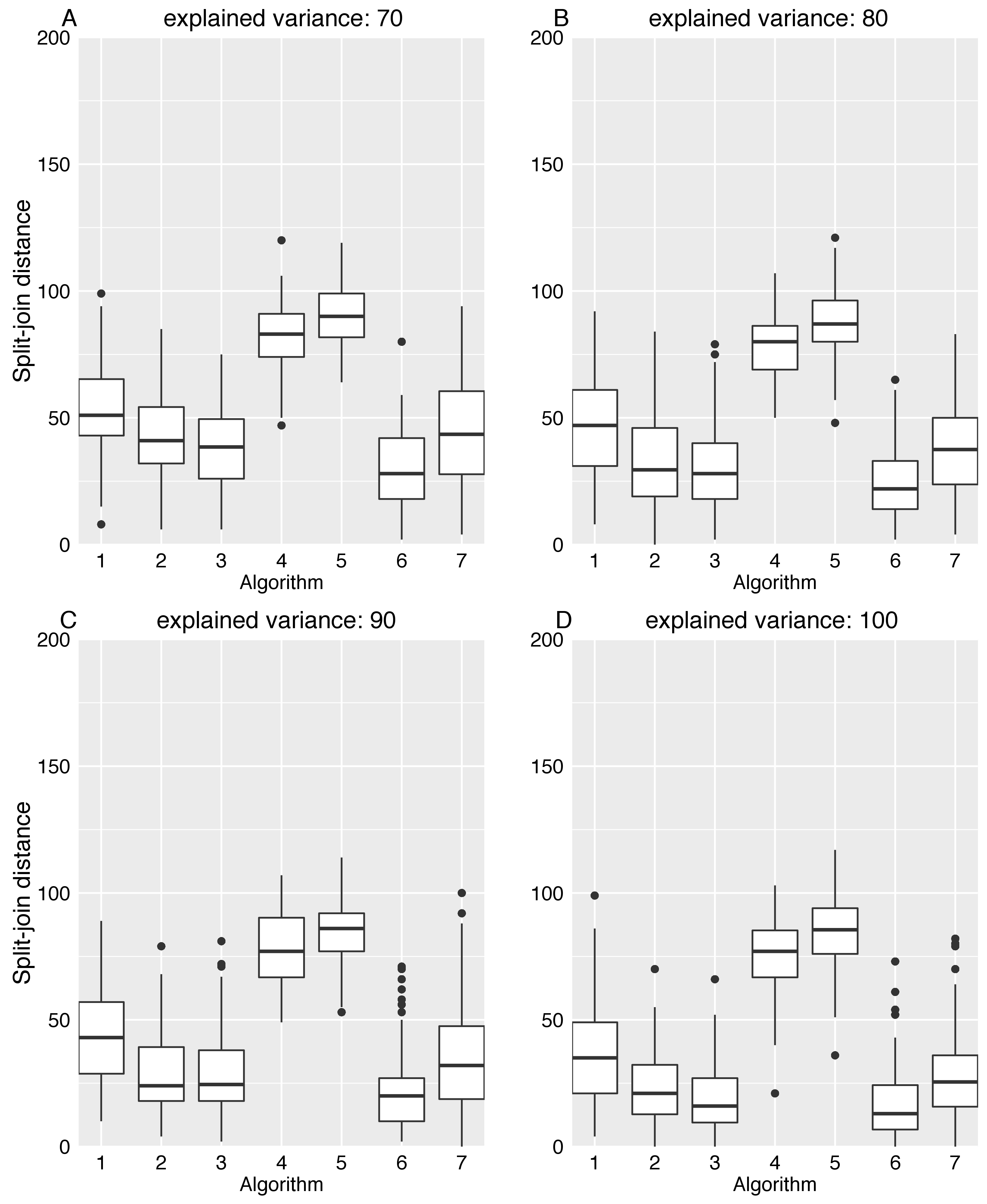

Supplement: S6 Fig — The split-join distance [Dongen, 2000] measures the extent to which two partitions are subpartitions of each other by means of accounting for their module overlap. The following algorithms for network module identification were used: “leading eigenvector” (1), “Louvain” directed (2), “Walktrap” (3), “fast greedy” (4), “leading eigenvector” (5), “Potts spin glass” (6), “Louvain” undirected (7). (A) ground truth network; (B-D) lsGCI network with variance explanations from 70%-90%; (E) GCI network. (TIFF) [file pone.0153105.s006.tiff]

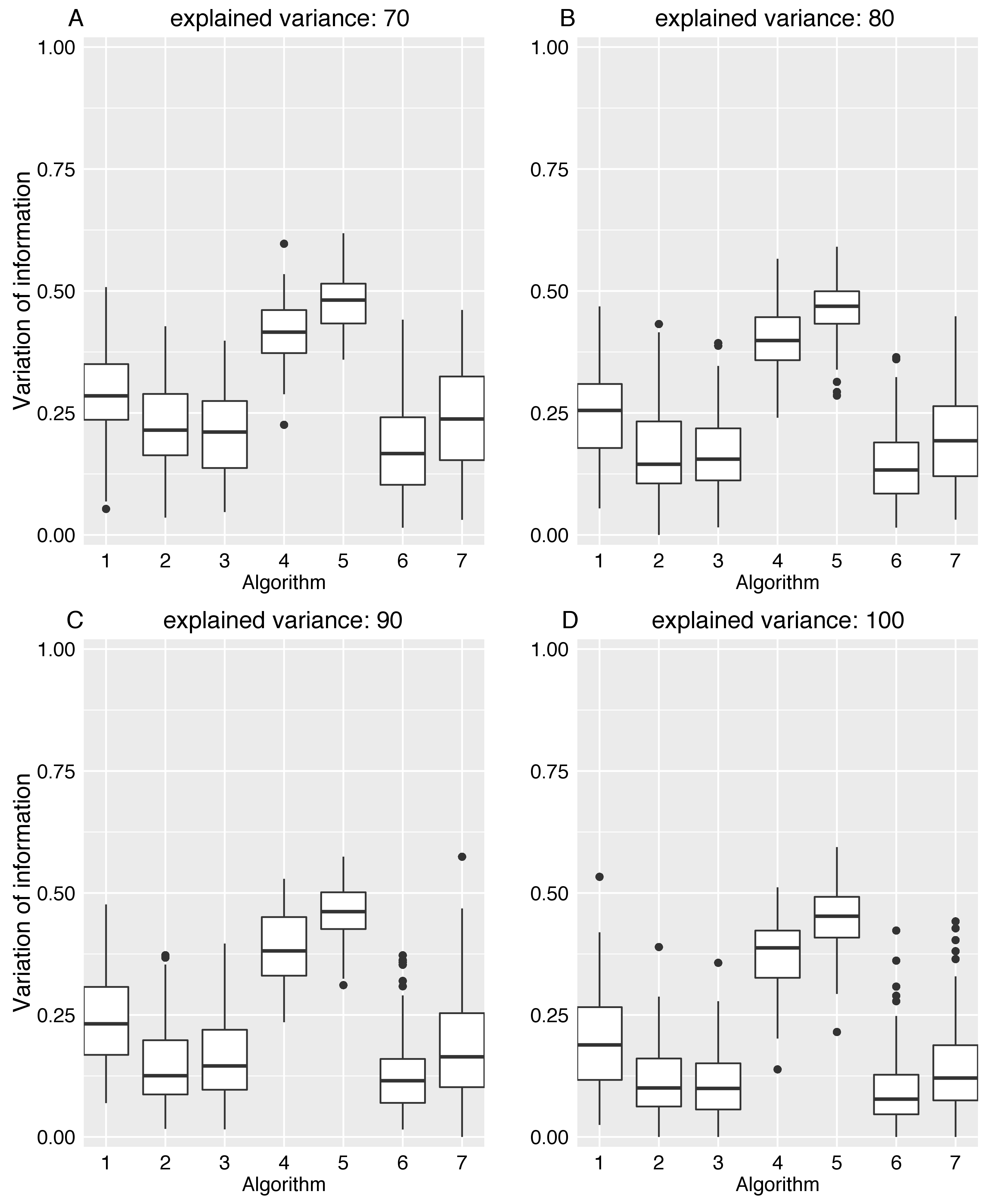

Supplement: S7 Fig — The variation of information [Meilă, 2007] compares two network partitions by measuring the change in their information (using entropy and mutual information) when one partition is converted to the other one. It is a metric on the space of network partitions and can consequently be used to calculate the distance of two partitions of the same network data. This measure does not depend on topological information of the input network as it relies only on module affiliations of vertices. The following algorithms for network module identification were used: “leading eigenvector” (1) “Louvain” directed (2) “Walktrap” (3), “fast greedy” (4), “leading eigenvector” (5), “Potts spin glass” (6), “Louvain” undirected (7). (A) ground truth network; (B-D) lsGCI network with variance explanations from 70%-90%; (E) GCI network. (TIFF) [file pone.0153105.s007.tiff]
