# Supplementary material for: A Multivariate Granger Causality Concept towards Full Brain Functional Connectivity
Source: PLoS One. 2016 Apr 11;11(4):e0153105. doi: 10.1371/journal.pone.0153105 (PMC4827851; doi:10.1371/journal.pone.0153105)
Supplement: S1 File — (PDF) [file pone.0153105.s008.pdf]

Supporting information references

- Dasgupta S, Papadimitriou CH, Vazirani U (2006): Algorithms 1st ed. McGrawHill.
- Dongen S (2000): Performance criteria for graph clustering and Markov cluster experiments. CWI (Centre for Mathematics and Computer Science), Amsterdam, The Netherlands.
- Fortunato S (2010): Community detection in graphs. Physics Reports 486:75–174.
- Hubert L, Arabie P (1985): Comparing partitions. Journal of Classification 2:193–218.
- Leicht EA, Holme P, Newman MEJ (2006): Vertex similarity in networks. Phys Rev E 73:026120.
- Leicht EA, Newman MEJ (2008): Community structure in directed networks. Phys Rev Lett 100:118703
- Levenshtein VI (1966): Binary codes capable of correcting deletions, insertions, and reversals. Soviet Physics-Doklady 10:707–710.
- Meilă M (2007): Comparing clusterings—an information based distance. Journal of Multivariate Analysis 98:873–895.
- Newman MEJ (2012): Communities, modules and large-scale structure in networks. Nat Phys 8:25–31.
- Newman MEJ, Girvan M (2004): Finding and evaluating community structure in networks. Phys Rev E 69:026113.
- Rand WM (1971): Objective Criteria for the Evaluation of Clustering Methods. Journal of the American Statistical Association 66:846–850.
- Rousseeuw PJ (1987): Silhouettes: A graphical aid to the interpretation and validation of cluster analysis. Journal of Computational and Applied Mathematics 20:53–65.
